# Variation in Tree Growth Increases With Global Warming

**DOI:** 10.1111/ele.70326

**Published:** 2026-01-27

**Authors:** Jingye Li, Fangliang He

**Affiliations:** ^1^ Department of Renewable Resources University of Alberta Edmonton Alberta Canada; ^2^ ECNU‐Alberta Joint Laboratory for Biodiversity Study, Tiantong National Station for Forest Ecosystem Research, School of Ecology and Environmental Sciences East China Normal University Shanghai China

**Keywords:** climate change impact, climate zones, ecological stability, forest resilience, growth‐safety tradeoff, hydraulic trait, Taylor's power law, tree radial growth, variance to mean relationship

## Abstract

Global warming is raising both climate and weather variability. However, how this tendency may destabilise forest ecosystems is poorly understood. Using a set of global tree‐ring data, we calculated the 5‐year variance and mean of tree growth rate over 1401–2010, and modelled the variance–mean relationship. We found that the global averaged variance increased much faster than the mean in the past century (+40.0% vs. +8.5%), and closely covaried with the accelerated global warming since the 1970s (*r* = 0.93). The exponent of tree‐level variance–mean power law was higher in wetter habitats and less drought‐resistant species, and has increased significantly under global warming, indicating an environment‐ and trait‐dependent growth‐safety tradeoff and a decreasing resistance to a warmer climate. Our study shows that global warming may have strongly destabilised tree growth and made forest dynamics less predictable, adding to the growing concern that global warming is jeopardising the functioning of forest ecosystems.

## Graphical abstract

In recent decades, considerable research has focused on the long‐term trend of tree growth rates under global warming, yet little attention has been paid to trends in high‐frequency tree growth variations—that is, year‐to‐year differences in growth rates. Our study reveals for the first time that, over the past century, the global average tree growth variation has increased by 40%, a far more pronounced shift than the 8.5% increase observed in long‐term mean tree growth rates. These findings indicate that global forest growth is becoming significantly more unstable, which may jeopardize the well‐functioning of forest ecosystems.

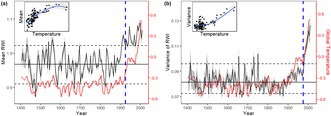

## 1 Introduction

Global warming elevates mean temperatures and also increases climate and weather variabilities (annual deviations from long‐term averages, and shorter‐term atmospheric changes, respectively) (Rahmstorf and Coumou 2011; AghaKouchak et al. 2020). These changes directly affect the performance of organisms and can significantly alter the tendency and variation (temporal uncertainty) of ecological processes. Tree growth is a fundamental process driving the dynamics and functioning of global forests. Quantifying tree growth responses to global warming is therefore critical for understanding and predicting the capacity of forests in providing ecosystem services, such as carbon sequestration, biodiversity maintenance, and climate change mitigation. However, previous studies predominantly focused on the temporal trend of tree growth rate, while largely neglecting the trend of growth variation.

Tree growth critically relies on a balanced energy (temperature and light) and water (precipitation) supply (Lambers et al. 2008). As global warming progresses and atmospheric CO_2_ concentration continues to rise, it has been widely noticed that tree growth has accelerated globally, especially in higher latitudes (McMahon et al. 2010; Pretzsch et al. 2014; Silva et al. 2016; D'Orangeville et al. 2018). Recently, evidence also shows that such growth acceleration may be weakening or even reversing in some regions, mainly due to the warming‐induced droughts and heatwaves, which suppressed growth and inflicted tree mortality (Allen et al. 2010; Peng et al. 2011; Tijerín‐Triviño et al. 2025). Associated with these changes in long‐term growth tendency, tree growth rate also fluctuates widely from year to year (Clark and Clark 1994). Such growth variation could increase as global climates become more extreme and environmental disturbances intensify (e.g., through air pollution, fires, pest outbreaks, and land use changes) (Rahmstorf and Coumou 2011). This potential increase of growth variation would be ecologically consequential as it indicates compromised individual tree health and carbon sequestration efficiency (Cailleret et al. 2019; DeSoto et al. 2020). This is because not only is less carbon assimilated during low‐growth events, but a greater proportion of the fixed carbon could be consumed in defending against the factors that caused those events—such as drought, heat, diseases, and pests—diverting carbon from wood production and storage. This individual‐level growth instability could escalate to destabilise population‐ and community‐level productivity and ultimately undermine the resilience of forest ecosystems and their functional roles (Isbell et al. 2009; Lloret et al. 2011; Cailleret et al. 2019; DeSoto et al. 2020).

Despite the ecological significance of both tree growth rate and its temporal variation, the latter has received far less attention than the former in previous studies. We don't know how growth variation changes at the global level as global warming progresses, nor do we know how it covaries with mean tree growth rate. Addressing this knowledge gap could help better understand the impacts of global warming (and other associated environmental changes) on tree growth and forest dynamics. Under recent global warming, particularly since the 1970s when warming intensified, the sensitivity of tree growth to temperature has decreased (Babst et al. 2019). We thus hypothesize that the increase in mean growth rate would have slowed down (IPCC 2021). Meanwhile, growth variation could exhibit an accelerating upward trend as excessive warming and droughts push trees toward their physiological limits, leading to a less stable tree growth process.

Numerous factors, both abiotic and biotic, affect tree growth and its variation. Previous studies showed that faster tree growth rates are associated with lower stress tolerance (to drought in particular) and therefore result in higher climate sensitivities (Eller et al. 2018; Bauman et al. 2022). The possible mechanism is that fast‐growing trees produce xylem conduits with larger diameters and thinner walls, which allow higher hydraulic conductivity but make the xylem structures more susceptible to damages caused by drought‐induced negative water pressure (Gleason et al. 2016; Eller et al. 2018). Such vulnerability could increase tree growth sensitivity to climate variability, thus increase variations in tree growth and ultimately weaken forest ecosystem stability (Cailleret et al. 2019; DeSoto et al. 2020). This positive relationship between mean tree growth rate and growth variation could also result if faster tree growth rates mean tree growth is more sensitive to favourable short‐term climatic anomalies, causing stronger growth variation. However, the opposite may also be true: faster‐growing trees may have larger reserves of non‐structural carbon and other nutrients (Carbone et al. 2013), which can buffer the negative impacts of extreme climate events and reduce growth variations (Hartmann and Trumbore 2016; Gessler et al. 2017). In both cases, the growth rate‐variation relationship could depend on species traits, which determine tree growth responses to environmental fluctuations (Rowland et al. 2021; Bauman et al. 2022). Despite that the tree‐level growth‐variation relationship has the potential to provide critical insights into the tradeoff between growth rate and stability, as well as the adaptability of forests to the changing environment, no effort has so far been made to quantify this relationship and its dependency on climates and species traits. Addressing this gap can help predict the response of forests to climate change and inform management practice.

In this study, we compiled a global tree‐ring dataset spanning from 1401 to 2010 to reconstruct the global histories of tree growth rate and variation, and modelled their relationship. Specifically, we aim to: (1) examine the respective global trends of mean tree growth rate and growth variation over the past 600 years, (2) quantify the effects of climate on mean tree growth rate and growth variation, and (3) model the variance–mean power law relationship for individual tree growth, and examine the effects of environmental conditions and species traits on the power‐law relationship.

## 2 Methods

### 2.1 Tree‐Ring Data

Tree‐ring data used in this study were compiled from the International Tree‐ring Data Bank (https://www.ncdc.noaa.gov). In the database, tree‐ring series are grouped into separate datasets, each containing trees from one location and one species (defined as one population). All datasets available by May 2022 were downloaded. We cleaned the datasets following the widely adopted method of Zhao et al. (2019), which included removing duplicated trees, standardising site and species names, correcting formatting errors (such as relocating erroneous stop markers to ensure data integrity and software readability), etc. We restricted our analysis to tree‐ring data falling in the period of 1401–2010 because the sample size in 1401 and 2010 is similar and is much reduced before 1401 and after 2010 (Figure S1). The cleaned data includes 208,267 series from 5361 populations (4251 unique sites) and 281 species across the world (Figure S1).

**FIGURE 1:**
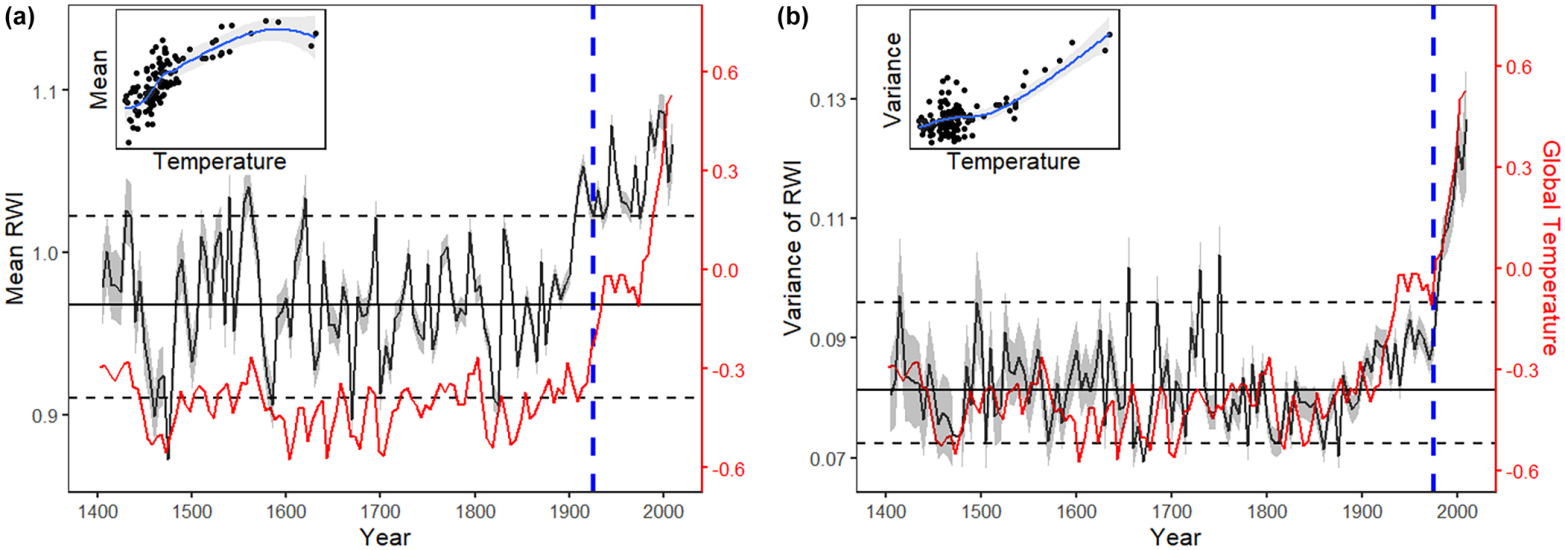
Global tree growth patterns over 1401–2010. (a) The 5‐year moving window mean of tree growth rates (RWI) as shown by the black curve. The gray zone is the ±1.96 standard error interval for the mean. (b) The associated temporal variance of tree growth rates. The dashed blue line indicates the year after which non‐stationarity was detected by the Dickey‐Fuller test. The solid horizontal lines are the stationary means of growth rates and variance, respectively, calculated over 1401–1900. The dashed horizontal lines are their 0.05 and 0.95 quantiles, respectively. These stationary means are the baselines of the growth rates and variance against which the increase in growth rate in 1925–2010 and variance in 1975–2010 are compared. Red lines are the global average temperature anomalies (with respect to 1961–1990 mean, which is 13.97°C). The inset panels show the relationships of growth mean and variance with the temperature anomalies, respectively.

Tree‐ring widths represent annual radial growth rates of trees. It is well recognised that raw ring‐width measurements inherently decrease with tree age, which could inflate or shadow any climate‐driven long‐term growth trend (Cook et al. 1990; Bowman et al. 2013). This confounding effect must be controlled before further analysis. To do that, it is a common practice to fit an age‐related ‘expected growth curve’ and divide the measured ring width by the expected curve (Cook et al. 1990; Babst et al. 2019). This process is known as ‘detrending’ or ‘standardising’, and the calculated dimensionless value is called Ring‐Width Index (RWI). There are various methods to fit the expected growth curve, among which the spline function, regional curve, and negative exponential function are most commonly used (Cook et al. 1990; Helama et al. 2004). To rule out potential biases caused by different detrending methods, all three types of growth curves were used to detrend the raw data and to reconstruct historical tree growth patterns in this study. We further tested two more detrending methods: (1) to fit the curve based on the constant basal area increment assumption (Biondi and Qeadan 2008) and (2) using raw growth data without detrending but controlling age effect in a mixed‐effects model. Details on these detrending sensitivity tests can be found in Supporting Information, Methods S1. In short, all five methods led to similar results, showing a significant increase in both mean and variance of growth rate from their historical baselines (*p* < 0.001), in line with the main findings (see Figure 1 in Results). The only exception was that the mean growth rate derived from the 2/3 spline detrending showed an insignificant trend (*p* > 0.05), which was expected given that it is a more aggressive detrending method that removes a larger portion of long‐term growth signals. Since the negative exponential function detrending has been known to be able to retain both long‐term and short‐term climatic signals, which are necessary for our study (Helama et al. 2004; Gedalof and Berg 2010), we chose this detrending method here. We calculated RWI by specifying ModNegExp in the *detrend.series* function provided by package *dplR* (Bunn 2008) of R software (https://r‐project.org). In addition to detrending, we evaluated additional factors that could influence tree‐ring data and potentially bias our results (Methods S1).

### 2.2 Climate Data

We compiled two sets of climate data. The first one was the global mean annual temperature (*MAT*_*g*_) covering the whole study period of 1401–2010 (Neukom et al. 2019). The second data, compiled from dataset *CRU‐TS4.04* (0.5° resolution; http://climexp.knmi.nl) for individual sites, included mean annual temperature (*MAT*), total annual precipitation (*TAP*), mean annual self‐calibrating Palmer Drought Severity Index (*scPDSI*), mean annual water vapour pressure (*VP*), mean annual relative humidity (*RH*), and mean annual water vapour pressure deficit (*VPD*). Here *RH* and *VPD* were calculated from temperature and water vapour pressure. These data spanned a shorter time period from 1901 to 2010.

### 2.3 Species Traits

We compiled leaf and wood economic spectrum traits from the *TRY* plant trait database (http://try‐db.org) (Kattge et al. 2011) to test possible associations of those traits important to growth and stress resistance with tree growth variance–mean relationships. The availability of the trait data for our 281 species varied, with 57 species having data of *specific leaf area* (*SLA*), 51 species having *leaf/sapwood area ratio* (*LSR*), 73 species having *negative xylem pressure at 50% conductivity loss* (*P*50), 162 species having *wood density* (*WD*), and 37 species having *stem conduit density* (*SCD*).

### 2.4 Temporal Trends of Mean and Variance of Tree Growth Rates

To reconstruct the historical trends of tree growth rate and growth variation, we calculated the mean and variance of individual tree's annual growth rates (the RWI values) using non‐overlapping 5‐year moving windows from 1401 to 2010 (Suvanto et al. 2016; Philipsen et al. 2018). The 5‐year window was selected to balance temporal resolution and high‐frequency noise suppression. We also tested other window lengths (3, 5, and 10 years) and found our main conclusions were robust to the test (Figure S2). For each 5‐year moving window, the mean and variance of growth rate (RWI) were respectively calculated for each tree and averaged into populational values. We then calculated the global mean and variance by averaging the populational values across the world for each 5‐year window (i.e., each population contributes one data point to the global averages). To determine the time when the mean and variance of tree growth rates have significantly deviated from their respective historical mean, we assessed the stationarity of the two time series using the Dickey‐Fuller test in R package *tseries* (Trapletti and Hornik 2023). Pearson's correlation coefficient was calculated between global mean temperature (*MAT*_*g*_) and the global average mean and variance of tree growth rates, respectively. To reveal tree growth patterns in different climatic environments, we also averaged the mean and variance for each of the five Köppen climate zones (*Tropical*, *Dry*, *Temperate*, *Continental*, *Polar*; Figure S1), classified by temperature and precipitation in 1951–2000 (Kottek et al. 2006). Note that in the calculations above, the upper and lower 0.01 quantiles of populational values were excluded to minimise possible bias caused by outliers.

Although the above correlation analysis assesses the overall global‐level relationship of tree growth (mean and variance, respectively) with temperature, the correlations could vary across climate zones and be affected by zone‐specific non‐climatic factors. To account for these climate zone‐related confounding effects, we modelled tree growth mean and variance on multiple climate factors using a linear mixed‐effects model by treating climate zone as a random effect. The fixed effects included temperature and water‐related climatic variables, calculated the same way the mean and variance of tree growth rates were calculated. These climatic variables included 5‐year mean: *MAT*, *MAT*_*g*_, *TAP*, *scPDSI*, *VP*, *RH*, and *VPD* and their 5‐year variance: *MAT*_*var*_, *MAT*_*gVar*_, *TAP*_*var*_, *scPDSI*_*var*_, *VP*_*var*_, *RH*_*var*_, and *VPD*_*var*_. The quadratic term of both *MAT*_*g*_ and *MAT* were also included, denoted as *MAT*_*g*_^2^ and *MAT* ^2^, to capture the nonlinear relationship between growth and temperature as observed in Figure 1. The linear mixed‐effects model is:

(1) $$y_{i}=\beta_{0}+\beta_{1}x1_{i}+\ldots +\beta_{k}{\mathrm{xk}}_{i}+u_{i}+\varepsilon_{i}$$

where $y_{i}$ is either the mean or variance of tree growth rate in year *i* (referring to the middle year of a five‐year window), ${\mathrm{xk}}_{i}$ is the *k* ^th^ climatic variable, $u_{i}$ is the random effect of climate zone, and $\varepsilon_{i}$ is the model residuals. Modelling was separately conducted for two different time spans: 1401–2010 when only *MAT*_*g*_, *MAT*_*g*_^2^ and *MAT*_*g*Var_ were used (because other variables were not available before 1901; see the data section above), and 1901–2010 when all climatic variables (regional average anomalies, relative to 1961–1990) were used. Note that climate anomalies rather than the raw measurements were used to ensure that the model captures the temporal rather than spatial growth‐climate relationship. All climatic variables were standardised to 0–1 by (*x*‐*x*_min_)/(*x*_max_‐*x*_min_) before modelling, to make their effect sizes comparable. Model selection was based on the AIC.

### 2.5 Modelling Tree Growth With Taylor's Variance–Mean Power Law

To examine the tree‐level relationship between the mean and variation of tree growth rates, we modelled tree growth data following Taylor's variance–mean power law (Taylor 1961):

(2) $$v=a\;\mu^{b}$$

where v and μ are variance and mean, respectively, while *a* and *b* are two coefficients, with exponent *b* being of particular interest as it captures the nature of the scaling relationship (Cohen and Xu 2015). Specifically, the relationship between mean and variance is isometric if *b* = 1, accelerating (superlinear) if *b* > 1, and decelerating (sublinear) if *b* < 1. Empirical data show *b* typically falls between 1 and 2 (Taylor 2019). In our study, the value of *b* indicates the degree of tree growth variation scales with tree growth rate. We did two analyses to model the Taylor power law. The first analysis was to fit the power law to growth variance–mean data for the entire study period of 1401–2010 but in multiple consecutive 50‐year windows, in order to examine the temporal changes in the mean–variance relationship. The second analysis was to model the relationship for the period of 1901–2010 in which tree growth rates and variance had non‐stationary increase and local climate data were also available, allowing us to examine the variability in the mean–variance relationship over space and across species. The power‐law fittings of the two analyses are described below.

1. We divided the time range of tree‐ring data into consecutive 50‐year periods (totaling 12 periods: 1411–1460, 1461–1510, …, 1961–2010; the first 10 years of 1401–1410 were excluded so that to make the last period ending in 2010). For each 50‐year period, any tree‐ring series that did not have full overlap with that period was excluded. Populations with fewer than 3 series were also removed. Within each of the twelve 50‐year periods, we first calculated tree growth mean and variance using 5‐year moving windows (i.e., with 4‐year overlap). We used a 4‐year overlapping window to fully leverage the information the tree‐ring data carries to increase the sample size for more robust model fitting: given the 50‐year period, a non‐overlapping approach would yield only 10 data points, which is insufficient for reliable fitting of the power law model. The power‐law model was then fitted to the variance and mean data of each 50‐year period. The fitting was conducted by applying the ordinary least‐squares method to the log‐transformed model (Equation 2). The exponent *b* values were first averaged within each population then averaged across all the populations to obtain a global *b* value for each of the 12 50‐year periods. A schematic graph for the averaging process can be found in the Supporting Information (Figure S3). The upper and lower 0.01 quantiles of populational mean exponents were removed to minimise the influence of outliers.
2. We fitted the same power‐law model to each tree‐ring series of 1901–2010 and calculated a mean exponent *b* for each population. We excluded tree‐ring series which covered shorter than 60 years over the period of 1901–2010 and populations with fewer than 3 series. Finally, a total of 155,364 tree‐ring series were modelled, which represented 4890 populations. The tree‐level exponents (*b*) were then averaged to obtain a mean exponent for each population. The upper and lower 0.01 quantiles of populational mean exponents were removed to minimise the influence of outliers, resulting in 4791 populational exponents. From these populational *b* values, we obtained an average *b* across the world and for each of the five climate zones.

### 2.6 Climate and Trait Dependency of the Power‐Law

First, to assess the potential temporal association between exponent *b* and global warming, we calculated Pearson's correlation coefficient between the 50‐year global average *b* and mean temperature.

Next, to examine the spatial variabilities in *b*, we first tested the climate‐zone level differences of populational *b* (for the period of 1901–2010) using Tukey's multiple comparison. We then modelled populational *b* using the local climates and species traits with a linear mixed‐effects model:

(3) $$b_{\mathrm{ij}}=\beta_{0}+\beta_{1}x1_{\mathrm{ij}}+\ldots \beta_{k}{\mathrm{xk}}_{\mathrm{ij}}+u_{i}+\varepsilon_{\mathrm{ij}}$$

where $b_{\mathrm{ij}}$ is the exponent for the *j* ^th^ population of the *i* ^th^ species, ${\mathrm{xk}}_{\mathrm{ij}}$ is the *k* ^th^ predictor (climate or traits), $u_{i}$ is species specific random effect describing the variation in *b* among populations within a species, and $\varepsilon_{\mathrm{ij}}$ is the model residuals. Climatic variables (*MAT*, *TAP*, *scPDSI*, *VP*, *RH*, *VPD*, *MAT*_*var*_, *TAP*_*var*_, *scPDSI*_*var*_, *VP*_*var*_, *RH*_*var*_, and *VPD*_*var*_) were mean values over 1901–2010. As the availability of the trait data varies among species, if the number of traits included in a model increases, the number of species (and populations) included in the model decreases (i.e., sample size decreases). Given this constraint, we built two models. The first model only included climatic variables, so that no species or population needs to be excluded. The second model included both climatic and trait variables. To fit this second model, we could not include all traits in one model because the availability of trait data varied across species. Instead, we used a two‐step approach. First, we fitted the model to each possible combination of traits plus all climatic variables. In the case where any of the traits in the combination was insignificant, that model was excluded. Models with fewer than 10 species were also excluded. Finally, among the models retained, the model with the lowest AIC was selected. All numerical predictors were standardised to 0–1 before modelling. Collinearity was checked and none was detected. Marginal and conditional *R* ^2^ (respectively denoted as $R_{m}^{2}$ and $R_{c}^{2}$) were calculated for the final models to quantify how much variation was explained by the fixed effects alone ($R_{m}^{2}$) or by the fixed and random effects together ($R_{c}^{2}$) (Nakagawa and Schielzeth 2013). A schematic plot for the above analysis can be found in the Supporting Information (Figure S4).

## 3 Results

### 3.1 Global Trends in Mean and Variance of Tree Growth Rate and Climatic Drivers

The Dickey‐Fuller test showed that the global mean and variance of tree growth rates as measured by ring width index were statistically stationary from 1401 to 1925 for the mean growth rate and 1401 to 1975 for the growth variance (Figure 1). They respectively increased significantly after 1925 and 1975 according to the test (*p* < 0.05), with the mean increasing by 8.5% and the variance increasing by 40.0% compared to their respective baseline means (Figure 1). Different from the mean growth rate (Figure 1a), there was a pronounced surge in the variance starting from the 1970s, with the variance increasing by 30.1% in just four decades (1971–1980 vs. 2001–2010) and still showing no sign of slowing down (Figure 1b). Similar patterns of mean and variance of tree growth rates, particularly the rapid increase of variance after the 1970s, were observed in all climate zones (Figure S5).

The observed global trends in tree growth mean and variance were strongly correlated with *MAT*_*g*_ over the past six centuries (Figure 1), with Pearson's correlation coefficient (*r*) of 0.80 between *MAT*_*g*_ and mean growth rate, and 0.73 between *MAT*_*g*_ and growth variance (1401–2010, *p* < 0.01). The surge in growth variance since 1975 was strongly correlated with the acceleration of global warming (*r* = 0.93 between variance and *MAT*_*g*_, *p* < 0.01, 1975–2010). It is also worth noting that, as global warming intensified, the increase of mean growth rates levelled off, while the increase of growth variance kept accelerating (Figure 1, insets).

The linear mixed‐effects model of the global growth patterns from 1401 to 2010 showed that, after controlling for differences among climate zones, both mean growth rate and growth variance maintained a significant positive relationship with temperature. Additionally, the model revealed that the second‐order term of temperature, *MAT*_*g*_^2^, has a negative relationship with mean growth rate but a positive relationship with growth variance, in line with the insets in Figure 1. For the period 1901–2010, *PDSI* was the most important predictor of mean tree growth rates, indicated by its largest effect size, followed by *MAT*, while *MAT* was the only and a highly significant predictor for tree growth variance (Table 1).

**TABLE 1:** Linear mixed‐effects models for quantifying the association of the mean and variance of tree growth rates with climatic variables. Coefficient (se) indicates the effect of a predictor and its related standard error. *R* ^2^ (m/c) indicates marginal and conditional *R* ^2^ for each model. *MAT*_*g*_, the global mean annual temperature; *MAT*_*g*_^2^, the squared *MAT*_*g*_; *MAT*, mean annual temperature; *scPDSI*, mean annual self‐calibrating Palmer Drought Severity Index.

Time	Response	Predictor	Coefficient (se)	*p*‐value	*R* ^2^(m/c)
1401–2010	Mean	*MAT*_*g*_	0.32 (0.03)	7.84E‐31	0.30/0.36
		*MAT*_*g*_^2^	−0.18 (0.03)	7.73E‐09	
	Variance	*MAT*_*g*_	0.04 (0.01)	5.13E‐06	0.11/0.82
		*MAT*_*g*_^2^	0.04 (0.01)	1.59E‐05	
1901–2010	Mean	*scPDSI*	0.17 (0.03)	2.77E‐07	0.25/0.29
		*MAT*	0.07 (0.02)	7.02E‐03	
	Variance	*MAT*	0.07 (0.01)	2.36E‐11	0.07/0.86

### 3.2 Individual Variance–Mean Power Law and Its Spatial and Temporal Variations

**FIGURE 2:**
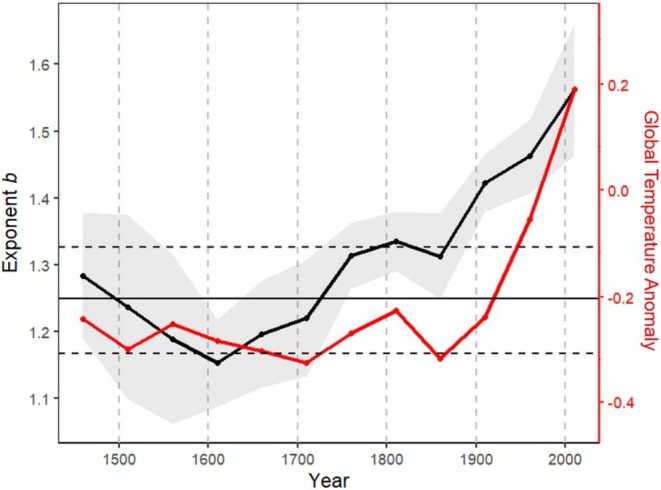
The global average exponent *b* of tree growth mean–variance power law increased over the past six hundred years (black curve). The gray zone represents ±1.96 standard error interval for the exponent *b*. The year on *x*‐axis is the end year of each 50‐year extent period over which the mean–variance power law was modelled. The horizontal black line is the baseline average of *b* (=1.25) over the period of 1401–1900, accompanied by the two dashed lines indicating the 0.05 and 0.95 quantiles. The red curve is the global mean temperature. The correlation between the exponent *b* and the mean temperature is *r* = 0.83.

**FIGURE 3:**
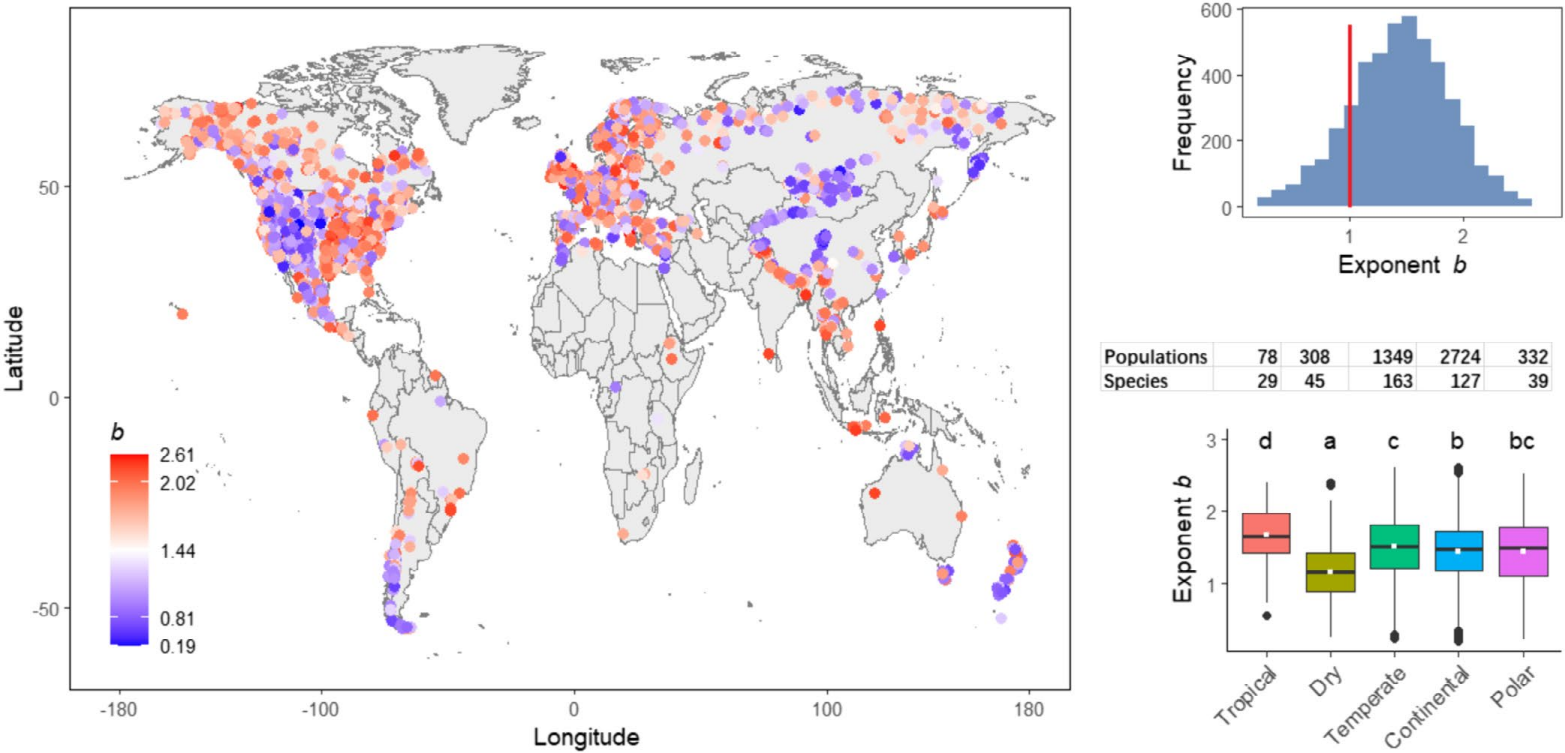
Global distribution of populational growth variance–mean power‐law exponent *b* for 1901–2010. The global average of *b* = 1.44. The right panels are the frequency distribution of *b* (with 84% of them having values > 1) and the Box plot of *b* across the five climate zones (white dots denote mean values, letters indicate the difference as tested by Tukey's multiple comparisons). The number of populations and species included in each climate zone are listed on top of each box.

Over the past six centuries, we observed a strong temporal trend in the growth variance–mean relationship, with the global exponent *b* increasing from a baseline mean of 1.25 to 1.56 in 2010 (Figure 2). This increase in *b* was closely correlated with temperature *MAT*_*g*_ with *r* = 0.83 over 1401–2010. For the period 1901–2010 in which tree growth rates and variance significantly increased (Figure 1), our tree‐level variance–mean relationship showed a median *R* ^2^ of 0.22, with 76% of trees exhibiting statistically significant relationships (*p* < 0.05) (Figure S6). The populational mean exponent had a global average value of 1.44 (± 0.02), with 84% of them larger than 1 (Figure 3), indicating that in this period tree growth variation overwhelmingly increased faster than the mean growth rate. At the regional scale, the climate zones *Tropical* and *Dry* had the highest and lowest mean *b* among all climate zones, respectively (Figure 3).

The 1901–2010 populational exponent *b* was significantly correlated with climates and species traits (Table 2). The linear mixed‐effects model explained 11% of the global variability in *b* when only climatic predictors were included, while 17% of the variability was explained when both climatic and trait variables were included (in this case trait data were only available for 3137 populations of 73 species; Table 2). The models showed that the exponent increased with air humidity (*VP*) and drought vulnerability (*P*50, of which a higher value indicates a lower resistance to drought) (Table 2).

**TABLE 2:** Linear mixed‐effects models between the exponent (*b*) of the mean–variance power‐law model and climate and trait variables. Coefficient (se) indicates the effect of a predictor and its related standard error. *R* ^2^ (m/c) indicates marginal and conditional *R* ^2^ for each model. *N.pop* is the number of populations (sample size) that were modelled, and *N.sp* is the number of species (the random effect). *P50* (*negative xylem pressure at 50% conductivity loss*) is the only trait selected by the model.

Response	Model	Predictor	Coefficient (se)	*p*‐value	*R* ^2^ (m/c)	*N.pop*	*N.sp*
*b*	climate only	*VP*	0.62 (0.10)	7.20E‐10	0.11/0.40	4791	256
		*VPD*_*var*_	0.31 (0.10)	1.20E‐03			
		*VP*_*var*_	0.21 (0.07)	2.30E‐03			
		*TAP*	0.11 (0.05)	2.44E‐02			
		*VPD*	−0.66 (0.07)	1.67E‐18			
	climate + trait	*VP*	0.74 (0.09)	7.67E‐17	0.17/0.40	3137	73
		*P50*	0.56 (0.13)	5.89E‐05			
		*TAP*_*var*_	0.32 (0.06)	1.93E‐07			
		*MAT*_*var*_	0.29 (0.06)	2.63E‐06			
		*VPD*	−0.43 (0.07)	1.17E‐09			

## 4 Discussion

Tree growth is a fundamental ecological process sustaining the global biosphere and carbon cycle (Vieira et al. 2005; Lambers et al. 2008; Pan et al. 2013). Understanding the response of tree growth to global warming is critical for informing climate mitigation practices. Unlike the warming‐stimulated tree growth, which is well documented globally (McMahon et al. 2010; Pretzsch et al. 2014; Silva et al. 2016; D'Orangeville et al. 2018), the response of tree growth variation to warming has been little explored. Our study shows that, over the past century, the global average 5‐year tree growth variance has increased nearly four times faster than the mean, particularly after the 1970s. We further found that the increase of mean growth rate with global temperature has levelled off in recent decades while the increase of variance accelerated (insets of Figure 1). However, it should be noted that the increase of mean growth rate in climate zone Polar did not level off, but instead accelerated (Figure S5). These findings suggest that, while there was a significant regional difference, global warming‐induced tree growth stimulation has in general weakened, and global tree growth has become less stable in recent decades. In addition, we found that at the individual level, tree growth variation also increased generally faster than mean growth rate, as indicated by the superlinear mean–variance relationship, with exceptions in drier regions such as the southwestern US and Central Asia (Figure 3).

While our analysis revealed a strong correlation between global mean tree growth rate and mean annual temperature, we caution to interpret this finding as evidence of a globally coherent tree growth stimulation by rising temperature, but rather evidence of a generally positive growth response to various warming‐related environmental changes. For example, tree growth in the eastern USA may have directly benefited from rising precipitation in the region due to warming‐induced shifts in atmospheric patterns (Alexander et al. 2021). A recent study showed that the main driver of global tree growth has shifted from temperature to water (Babst et al. 2019), which is in line with our finding that *scPDSI* emerged to be the most important predictor for mean tree growth rate in the last century (Table 1). Additionally, most trees in our dataset are from boreal forests, where tree growth is believed to respond more positively to global warming (McMahon et al. 2010; Pretzsch et al. 2014; Silva et al. 2016; D'Orangeville et al. 2018). However, as shown in our climate‐zone level analysis (Figure S5), although the largest growth increases occurred in *Polar* and *Continental* zones, positive tree growth trends can be found in all climate zones, including *Dry* zone.

The global decline in warming‐related tree growth and the continued increase of growth variance (Figure 1 insets) are further confirmed by the positive and negative terms of *MAT*_*g*_^2^ in the climate‐growth models (Table 1). These results reflect an overall unfavourable environmental shift for tree growth worldwide. The less‐dense data points at high temperatures shown in the insets of Figure 1 indicate that this growth pattern is a relatively recent phenomenon. They occurred probably because tree growth cannot effectively adapt to the excessive warming (Aguirre‐Gutiérrez et al. 2025), which exacerbates water deficits and offsets the earlier beneficial effects of warmer climates. This finding aligns with recent observations of decreasing forest growth and rising tree mortality on the global scale (Peng et al. 2011; Tijerín‐Triviño et al. 2025). These unfavourable environmental changes could also make living trees more sensitive to interannual variations in climate factors, especially precipitation (Babst et al. 2019; Peltier et al. 2022), thus elevating the interannual variation in tree growth rate. Finally, though not tested in this study, this increased tree growth variation might also be partially explained by other global change stressors, e.g., pests and diseases, acid rain, fires and land use change (Trumbore et al. 2015).

The overwhelmingly‐superlinear power‐law relationship (i.e., *b* > 1) means that when trees grow faster, they also vary more in growth rate. An increase in tree growth variation is often considered as a sign of weakening stress resistance and tree vigour (Lloret et al. 2011; Cailleret et al. 2019), making individual trees more vulnerable to extreme climates and various disturbances, subsequently increasing tree mortality (Trumbore et al. 2015; DeSoto et al. 2020). This result is in line with studies showing that the warming‐induced growth acceleration can reduce the lifespan of trees (Brienen et al. 2020). Compared to previous research that described this growth‐safety tradeoff with the growth‐lifespan relationship, for the first time we studied this tradeoff in living trees using the tree growth's mean–variance power law. Our findings indicate that the stimulation of tree growth by warming comes at the expense of growth stability and drought resistance. However, we would caution the inference about growth‐resistance tradeoff from the superlinear mean–variance relationship, because increased tree growth variation could also be caused by other reasons, such as increased growth sensitivity to favourable environment. Also, as drought could cause a legacy effect whereby those heavily‐impacted trees may enter a long low‐growth period (Cavin and Jump 2017; DeSoto et al. 2020), this could also reduce growth variance instead of increasing it.

Furthermore, our results indicate that this growth‐safety tradeoff varies significantly across species and climatic zones. As shown in Table 2, the hydraulic trait *P50* was significantly associated with the power law's exponent *b*. It is well recognised that species with less negative *P50* tend to grow faster but are more vulnerable to drought (Gleason et al. 2016). Therefore, faster‐growing species tend to be more susceptible to hydraulic system weakening induced by rapid growth, which further increases their sensitivity to climatic variation. This ultimately leads to a faster rise in growth variance with mean growth rate, and thus a larger exponent *b*. It has also been observed that trees growing in more humid climates tend to adopt a growth strategy that prioritises faster growth over building more robust hydraulic traits (Cavin and Jump 2017). This may explain why the effects of annual precipitation and water vapour pressure on exponent *b* are positive, while the effect of water vapour pressure deficit is negative. It also explains why the *b* is low in climatic zone *Dry* (Figure 3). In short, trees in drier habitats might inherently be more resistant to droughts, thus less susceptible to the fast growth‐induced weakening of hydraulic systems. Finally, the observation that the global exponent *b* changed from a baseline of 1.25 before global warming to 1.56 in the most recent decade suggests that under global warming, for each unit increase in growth rate, the increase in growth variation is becoming larger. In other words, an acceleration in growth comes at the cost of a greater loss of growth stability. In addition, the above findings may also suggest that the spatial and temporal variations in the mean–variance relationship of tree growth be driven by different climatic factors, with water being more important for spatial variation in *b* (Table 2) and temperature for temporal trends in *b* (Figure 2). However, these results should be interpreted with caution, as a positive correlation does not guarantee a causal relationship. In addition to precipitation and temperature, other abiotic and biotic factors not included in this study could also play a role.

Tree‐ring studies could be biased by various factors, such as sampling bias (e.g., toward large and more climate‐sensitive trees), detrending bias (including different detrending methods and end bias), and changing sample size or tree age over time (Bowman et al. 2013; Duchesne et al. 2019; Babst et al. 2019). We tested the effects of these common confounding factors and the results show the observed tree growth patterns in Figure 1 are robust (see Supporting Information, Methods S1). Still, there remain some caveats to clarify. First, it is necessary to emphasise that we used the ring width index, rather than raw tree‐ring widths, to represent tree growth rates in this study. Consequently, the interpretation of growth acceleration is on a relative scale. For example, although the *Polar* zone exhibited a greater relative magnitude of growth acceleration than the *Tropical* zone (Figure S5), the absolute growth rate is typically higher in tropical forests, where trees generally grow much faster. Second, while our results indicate that global warming is closely correlated with the observed upward growth trend and increased growth variance (Figure 1), we acknowledge that other global change factors may also influence these trends. For example, land use changes could very likely contribute to the increased tree growth variance by directly disturbing growth and altering competitive dynamics in forests (Cramer et al. 2008). Furthermore, natural forest succession, fire, diseases, acid rain, CO_2_ fertilisation, and nitrogen deposition could also play a role (Cook et al. 1990; Trumbore et al. 2015). Third, not all trees are amenable to tree‐ring study, especially those in warm and humid climates, as they don't typically form clear growth rings. As a result, the ITRDB tree‐ring data are less represented by tropical trees (Babst et al. 2019). We thus suggest not extrapolating the findings of our study to species or areas beyond the ITRDB data coverage.

## 5 Conclusion

Our findings that tree growth variation increased much faster than mean growth rate under global warming (especially since the 1970s) suggest that global warming has increasingly destabilised global forests and jeopardised their roles in supporting global life systems. The changing variance–mean power law with climate and species traits further suggests that trees from different environments and species have different abilities to maintain their growth stability. This study for the first time quantifies the global dynamics of tree growth variation and explores the causes. To better confront global change, we argue that future research should put more emphasis on understanding the temporal uncertainties in tree growth and their drivers.

## Author Contributions

Fangliang He and Jingye Li conceived the study. Jingye Li compiled the data, conducted the analysis, and wrote the first draft of the manuscript, which was revised by Fangliang He and Jingye Li.

## Funding

This work was supported by the Natural Sciences and Engineering Research Council of Canada and the Alberta Land Institute. Jingye Li also graciously acknowledges the Oversea Study Program of Guangzhou Elite Project (Grant: S.J.[2019] No. 2).

## Supporting information


**Table S1:** Linear mixed‐effects model for the responses of temporal mean and variance of tree growth rates (in mm) to temperature. Coefficient (se) indicates the effect (slope) of a predictor and its related standard error.
**Figure S1:** Geographical distribution (upper panel) and sample size (lower panel) of the tree‐ring data set. In the upper panel, colours denote different Köppen climate zones. In the lower panel the *y*‐axis denotes the number of populations. The black horizontal line denotes the sample size in 1401 which is 684 populations (while the number of populations in 2010 is 693).
**Figure S2:** Temporal trends of the global mean and variance of tree growth rates. Calculated using the same data and detrending method as in Figure 1, but with a different moving window length of 3 years (a, b) and 10 years (c, d).
**Figure S3:** Schematic plot showing the averaging process of tree‐level exponents from Taylor's power law models (see Methods for details).
**Figure S4:** Schematic plot showing the analysis to investigate the spatial and temporal regulators of the mean–variance relationship in tree growth rates (see Methods for details).
**Figure S5:** Temporal trends of the mean (a) and variance (b) of tree growth rates in different climate zones. Coloured solid lines are localised smooth regression lines. The thick blue lines denote averages among individual climate zones. Shaded area represents ±1.96 standard error interval. The vertical dashed blue lines denote year 1925 and 1975 for tree growth mean and variance, respectively.
**Figure S6:** Frequency distributions of the *p*‐value (a) and *R* ^2^ (b) for the tree‐level Taylor's power law model. The red line in (b) denotes the median value.
**Figure S7:** Temporal trends of the global mean and variance of tree growth rates. Calculated from tree‐ring series detrended by a 300‐year spline function (see Methods S1). The vertical dashed blue lines denote year 1925 and 1975 for mean and variance, respectively. Shaded area represents ±1.96 standard error interval. The *R* ^2^ values show the correlation between the growth mean or variance and that in Figure 1.
**Figure S8:** Temporal trends of the global mean and variance of tree growth rates. Calculated from tree‐ring series detrended by the 2/3 spline function. The vertical dashed blue lines denote year 1925 and 1975 for mean and variance, respectively. Shaded area represents ±1.96 standard error interval. The *R* ^2^ values show the correlation between the growth mean or variance and that in Figure 1.
**Figure S9:** Temporal trends of the global mean and variance of tree growth rates. Calculated from tree‐ring series detrended by the regional curve standardisation. The vertical dashed blue lines denote year 1925 and 1975 for mean and variance, respectively. Shaded area represents ±1.96 standard error interval. The *R* ^2^ values show the correlation between the growth mean or variance and that in Figure 1.
**Figure S10:** Temporal trends of the global mean and variance of tree growth rates. Calculated from tree‐ring series detrended by constant basal area increment detrending. The vertical dashed blue lines denote year 1925 and 1975 for mean and variance, respectively. Shaded area represents ±1.96 standard error interval. The *R* ^2^ value shows the correlation between the growth mean or variance and that in Figure 1.
**Figure S11:** Temporal trends of the global mean and variance of tree growth rates. Calculated from tree‐ring series fully covering 1801–2000 (no change in data coverage and sample size over this 200‐year period). The vertical dashed blue lines denote year 1925 and 1975 for mean and variance, respectively. Shaded area represents ±1.96 standard error interval. The *R* ^2^ values indicate the correlation between the growth mean or variance and that in Figure 1 in the same period of coverage time (1801–2000).
**Figure S12:** Temporal trends of the global mean and variance of tree growth rates. Calculated from tree‐ring series end before 1900 (for mean) and 1970 (for variance) (see Methods S1). Shaded area represents ±1.96 standard error interval.
**Figure S13:** Temporal trends of the global variance of tree growth rates. The variance was calculated for different age groups of trees separately. Each curve represents one age group. There are 10 groups in total. The red line denotes average values across all groups and each yellow point represents one 5‐year window over which growth variance was calculated. The vertical dashed blue line denotes year 1975.
**Figure S14:** Temporal trends of the global mean and variance of tree growth rates. Calculated from the ITRDB tree‐ring data that were originally collected for climate‐reconstruction studies (*upper panels*) and for other more general dendrochronological studies (*lower panels*). The global distributions of the two groups are displayed on the maps on the right, showing a fair global coverage. The vertical dashed blue lines denote year 1925 and 1975 for mean and variance, respectively. Shaded area represents ±1.96 standard error interval. The *R* ^2^ values show the correlation of the mean growth rate and variance with those in Figure 1.
**Figure S15:** Temporal trends of the global mean and variance of tree growth rates. Calculated from the group of trees represented by single series (*upper panels*) and the group of trees that may be represented by multiple series (*lower panels*). The vertical dashed blue lines denote year 1925 and 1975 for mean and variance, respectively. Shaded area represents ±1.96 standard error interval. The *R* ^2^ values show the correlation of the mean growth rate and variance with those in Figure 1.

## Data Availability

The data and codes that support the findings of this study are available at: https://doi.org/10.6084/m9.figshare.30882446.

## References

[ele70326-bib-0001] AghaKouchak, A. , F. Chiang , L. S. Huning , et al. 2020. “Climate Extremes and Compound Hazards in a Warming World.” Annual Review of Earth and Planetary Sciences 48, no. 1: 519–548.

[ele70326-bib-0002] Aguirre‐Gutiérrez, J. , S. Díaz , S. W. Rifai , et al. 2025. “Tropical Forests in the Americas Are Changing Too Slowly to Track Climate Change.” Science 387, no. 6738: eadl5414. 40048518 10.1126/science.adl5414

[ele70326-bib-0003] Alexander, H. D. , C. Siegert , J. S. Brewer , et al. 2021. “Mesophication of Oak Landscapes: Evidence, Knowledge Gaps, and Future Research.” Bioscience 71, no. 5: 531–542.

[ele70326-bib-0004] Allen, C. D. , A. K. Macalady , H. Chenchouni , et al. 2010. “A Global Overview of Drought and Heat‐Induced Tree Mortality Reveals Emerging Climate Change Risks for Forests.” Forest Ecology and Management 259, no. 4: 660–684.

[ele70326-bib-0005] Babst, F. , O. Bouriaud , B. Poulter , V. Trouet , M. P. Girardin , and D. C. Frank . 2019. “Twentieth Century Redistribution in Climatic Drivers of Global Tree Growth.” Science Advances 5: eaat4313. 30746436 10.1126/sciadv.aat4313 PMC6357745

[ele70326-bib-0006] Bauman, D. , C. Fortunel , L. A. Cernusak , et al. 2022. “Tropical Tree Growth Sensitivity to Climate Is Driven by Species Intrinsic Growth Rate and Leaf Traits.” Global Change Biology 28, no. 4: 1414–1432. 34741793 10.1111/gcb.15982

[ele70326-bib-0007] Biondi, F. , and F. Qeadan . 2008. “A Theory‐Driven Approach to Tree‐Ring Standardization: Defining the Biological Trend From Expected Basal Area Increment.” Tree‐Ring Research 64, no. 2: 81–96.

[ele70326-bib-0008] Bowman, D. M. , R. J. Brienen , E. Gloor , O. L. Phillips , and L. D. Prior . 2013. “Detecting Trends in Tree Growth: Not So Simple.” Trends in Plant Science 18, no. 1: 11–17. 22960000 10.1016/j.tplants.2012.08.005

[ele70326-bib-0009] Brienen, R. J. , L. Caldwell , L. Duchesne , et al. 2020. “Forest Carbon Sink Neutralized by Pervasive Growth‐Lifespan Trade‐Offs.” Nature Communications 11, no. 1: 4241. 10.1038/s41467-020-17966-z PMC7479146 32901006

[ele70326-bib-0010] Bunn, A. G. 2008. “A Dendrochronology Program Library in R (dplR).” Dendrochronologia 26, no. 2: 115–124.

[ele70326-bib-0011] Cailleret, M. , V. Dakos , S. Jansen , et al. 2019. “Early‐Warning Signals of Individual Tree Mortality Based on Annual Radial Growth.” Frontiers in Plant Science 9: 1964. 30713543 10.3389/fpls.2018.01964 PMC6346433

[ele70326-bib-0012] Carbone, M. S. , C. I. Czimczik , T. F. Keenan , et al. 2013. “Age, Allocation and Availability of Nonstructural Carbon in Mature Red Maple Trees.” New Phytologist 200, no. 4: 1145–1155. 24032647 10.1111/nph.12448

[ele70326-bib-0013] Cavin, L. , and A. S. Jump . 2017. “Highest Drought Sensitivity and Lowest Resistance to Growth Suppression Are Found in the Range Core of the Tree *Fagus sylvatica* L. Not the Equatorial Range Edge.” Global Change Biology 23, no. 1: 362–379. 27298138 10.1111/gcb.13366

[ele70326-bib-0015] Clark, D. A. , and D. B. Clark . 1994. “Climate‐Induced Annual Variation in Canopy Tree Growth in a Costa Rican Tropical Rain Forest.” Journal of Ecology 82: 865–872.

[ele70326-bib-0016] Cohen, J. E. , and M. Xu . 2015. “Random Sampling of Skewed Distributions Implies Taylor's Power Law of Fluctuation Scaling.” Proceedings of the National Academy of Sciences 112, no. 25: 7749–7754. 10.1073/pnas.1503824112 PMC4485080 25852144

[ele70326-bib-0017] Cook, E. R. , K. Briffa , S. Shiyatov , and V. Mazepa . 1990. Methods of Dendrochronology: Applications in the Environmental Sciences, edited by E. R. Cook and L. A. Kairiukstis , vol. 3, 98–123. Springer Science & Business Media.

[ele70326-bib-0018] Cramer, V. A. , R. J. Hobbs , and R. J. Standish . 2008. “What's New About Old Fields? Land Abandonment and Ecosystem Assembly.” Trends in Ecology & Evolution 23, no. 2: 104–112. 18191278 10.1016/j.tree.2007.10.005

[ele70326-bib-0019] DeSoto, L. , M. Cailleret , F. Sterck , et al. 2020. “Low Growth Resilience to Drought Is Related to Future Mortality Risk in Trees.” Nature Communications 11, no. 1: 545. 10.1038/s41467-020-14300-5 PMC6987235 31992718

[ele70326-bib-0020] D'Orangeville, L. , D. Houle , L. Duchesne , R. P. Phillips , Y. Bergeron , and D. Kneeshaw . 2018. “Beneficial Effects of Climate Warming on Boreal Tree Growth May Be Transitory.” Nature Communications 9, no. 1: 3213. 10.1038/s41467-018-05705-4 PMC6086880 30097584

[ele70326-bib-0021] Duchesne, L. , D. Houle , R. Ouimet , L. Caldwell , M. Gloor , and R. Brienen . 2019. “Large Apparent Growth Increases in Boreal Forests Inferred From Tree‐Rings Are an Artefact of Sampling Biases.” Scientific Reports 9, no. 1: 6832. 31048703 10.1038/s41598-019-43243-1 PMC6497877

[ele70326-bib-0022] Eller, C. , F. Barros , P. Bittencourt , L. Rowland , M. Mencuccini , and R. Oliveira . 2018. “Xylem Hydraulic Safety and Construction Costs Determine Tropical Tree Growth.” Plant, Cell & Environment 41, no. 3: 548–562. 10.1111/pce.13106 29211923

[ele70326-bib-0055] Gedalof, Z. , and A. A. Berg . 2010. “Tree Ring Evidence for Limited Direct CO2 Fertilization of Forests Over The 20th Century: Limited CO2 Fertilization of Forests.” Global Biogeochemical Cycles 24, no. 3: GB3027.

[ele70326-bib-0024] Gessler, A. , M. Schaub , and N. G. McDowell . 2017. “The Role of Nutrients in Drought‐Induced Tree Mortality and Recovery.” New Phytologist 214, no. 2: 513–520. 27891619 10.1111/nph.14340

[ele70326-bib-0025] Gleason, S. M. , M. Westoby , S. Jansen , et al. 2016. “Weak Tradeoff Between Xylem Safety and Xylem‐Specific Hydraulic Efficiency Across the World's Woody Plant Species.” New Phytologist 209, no. 1: 123–136. 26378984 10.1111/nph.13646

[ele70326-bib-0026] Hartmann, H. , and S. Trumbore . 2016. “Understanding the Roles of Nonstructural Carbohydrates in Forest Trees–From What We Can Measure to What We Want to Know.” New Phytologist 211, no. 2: 386–403. 27061438 10.1111/nph.13955

[ele70326-bib-0027] Helama, S. , M. Lindholm , M. Timonen , and M. Eronen . 2004. “Detection of Climate Signal in Dendrochronological Data Analysis: A Comparison of Tree‐Ring Standardization Methods.” Theoretical and Applied Climatology 79: 239–254.

[ele70326-bib-0028] IPCC . 2021. AR6 Climate Change 2021: The Physical Science Basis. Intergovermental Panel of Climate Change.

[ele70326-bib-0029] Isbell, F. I. , H. W. Polley , and B. J. Wilsey . 2009. “Biodiversity, Productivity and the Temporal Stability of Productivity: Patterns and Processes.” Ecology Letters 12, no. 5: 443–451. 19379138 10.1111/j.1461-0248.2009.01299.x

[ele70326-bib-0030] Kattge, J. , S. Diaz , S. Lavorel , et al. 2011. “TRY‐a Global Database of Plant Traits.” Global Change Biology 17: 2905–2935.

[ele70326-bib-0032] Kottek, M. , J. Grieser , C. Beck , B. Rudolf , and F. Rubel . 2006. “World Map of the Köppen‐Geiger Climate Classification Updated.” Meteorologische Zeitschrift 15: 259–263.

[ele70326-bib-0033] Lambers, H. , F. S. Chapin , and T. L. Pons . 2008. Plant Physiological Ecology. Springer.

[ele70326-bib-0034] Lloret, F. , E. G. Keeling , and A. Sala . 2011. “Components of Tree Resilience: Effects of Successive Low‐Growth Episodes in Old Ponderosa Pine Forests.” Oikos 120, no. 12: 1909–1920.

[ele70326-bib-0035] McMahon, S. M. , G. G. Parker , and D. R. Miller . 2010. “Evidence for a Recent Increase in Forest Growth.” Proceedings of the National Academy of Sciences 107, no. 8: 3611–3615. 10.1073/pnas.0912376107 PMC2840472 20133710

[ele70326-bib-0036] Nakagawa, S. , and H. Schielzeth . 2013. “A General and Simple Method for Obtaining R2 From Generalized Linear Mixed‐Effects Models.” Methods in Ecology and Evolution 4, no. 2: 133–142.

[ele70326-bib-0037] Neukom, R. , L. A. Barboza , M. P. Erb , et al. 2019. “Consistent Multi‐Decadal Variability in Global Temperature Reconstructions and Simulations Over the Common Era.” Nature Geoscience 12: 643–649. 10.1038/s41561-019-0400-0 PMC6675609 31372180

[ele70326-bib-0039] Pan, Y. , R. A. Birdsey , O. L. Phillips , and R. B. Jackson . 2013. “The Structure, Distribution, and Biomass of the World's Forests.” Annual Review of Ecology, Evolution, and Systematics 44, no. 1: 593–622.

[ele70326-bib-0040] Peltier, D. M. , W. R. Anderegg , J. S. Guo , and K. Ogle . 2022. “Contemporary Tree Growth Shows Altered Climate Memory.” Ecology Letters 25, no. 12: 2663–2674. 36257775 10.1111/ele.14130

[ele70326-bib-0041] Peng, C. , Z. Ma , X. Lei , et al. 2011. “A Drought‐Induced Pervasive Increase in Tree Mortality Across Canada's Boreal Forests.” Nature Climate Change 1, no. 9: 467–471.

[ele70326-bib-0042] Philipsen, L. J. , D. W. Pearce , and S. B. Rood . 2018. “Hydroclimatic Drivers of the Growth of Riparian Cottonwoods at the Prairie Margin: River Flows, River Regulation and the Pacific Decadal Oscillation.” Dendrochronologia 51: 82–91.

[ele70326-bib-0043] Pretzsch, H. , P. Biber , G. Schütze , E. Uhl , and T. Rötzer . 2014. “Forest Stand Growth Dynamics in Central Europe Have Accelerated Since 1870.” Nature Communications 5, no. 1: 4967. 10.1038/ncomms5967 PMC4175583 25216297

[ele70326-bib-0044] Rahmstorf, S. , and D. Coumou . 2011. “Increase of Extreme Events in a Warming World.” Proceedings of the National Academy of Sciences 108, no. 44: 17905–17909. 10.1073/pnas.1101766108 PMC3207670 22025683

[ele70326-bib-0045] Rowland, L. , R. S. Oliveira , P. R. Bittencourt , et al. 2021. “Plant Traits Controlling Growth Change in Response to a Drier Climate.” New Phytologist 229, no. 3: 1363–1374. 32981040 10.1111/nph.16972

[ele70326-bib-0046] Silva, L. C. , G. Sun , X. Zhu‐Barker , Q. Liang , N. Wu , and W. R. Horwath . 2016. “Tree Growth Acceleration and Expansion of Alpine Forests: The Synergistic Effect of Atmospheric and Edaphic Change.” Science Advances 2, no. 8: e1501302. 27652334 10.1126/sciadv.1501302 PMC5020709

[ele70326-bib-0047] Suvanto, S. , P. Nöjd , H. M. Henttonen , E. Beuker , and H. Mäkinen . 2016. “Geographical Patterns in the Radial Growth Response of Norway Spruce Provenances to Climatic Variation.” Agricultural and Forest Meteorology 222: 10–20.

[ele70326-bib-0048] Taylor, L. R. 1961. “Aggregation, Variance and the Mean.” Nature 189: 732–735.

[ele70326-bib-0049] Taylor, R. A. 2019. Taylor's Power Law: Order and Pattern in Nature. Academic Press.

[ele70326-bib-0050] Tijerín‐Triviño, J. , E. R. Lines , M. A. Zavala , et al. 2025. “Forest Productivity Decreases in Response to Recent Changes in Vegetation Structure and Climate in the Latitudinal Extremes of the European Continent.” Global Ecology and Biogeography 34, no. 2: e70011.

[ele70326-bib-0051] Trapletti, A. , and K. Hornik . 2023. “tseries: Time Series Analysis and Computational Finance (Version 0.10‐55) [R Package].” https://CRAN.R‐project.org/package=tseries.

[ele70326-bib-0052] Trumbore, S. , P. Brando , and H. Hartmann . 2015. “Forest Health and Global Change.” Science 349, no. 6250: 814–818. 26293952 10.1126/science.aac6759

[ele70326-bib-0053] Vieira, S. , S. Trumbore , P. B. Camargo , et al. 2005. “Slow Growth Rates of Amazonian Trees: Consequences for Carbon Cycling.” Proceedings of the National Academy of Sciences 102, no. 51: 18502–18507. 10.1073/pnas.0505966102 PMC1310511 16339903

[ele70326-bib-0054] Zhao, S. , N. Pederson , L. D'Orangeville , et al. 2019. “The International Tree‐Ring Data Bank (ITRDB) Revisited: Data Availability and Global Ecological Representativity.” Journal of Biogeography 46, no. 2: 355–368.

