# Supplementary material for: Variation in Tree Growth Increases With Global Warming
Source: Ecol Lett. 2026 Jan 27;29(2):e70326. doi: 10.1111/ele.70326 (PMC12843879; doi:10.1111/ele.70326)
Supplement: Supplementary file 1 — Table S1: Linear mixed‐effects model for the responses of temporal mean and variance of tree growth rates (in mm) to temperature. Coefficient (se) indicates the effect (slope) of a predictor and its related standard error. Figure S1: Geographical distribution (upper panel) and sample size (lower panel) of the tree‐ring data set. In the upper panel, colours denote different Köppen climate zones. In the lower panel the y‐axis denotes the number of populations. The black horizontal line denotes the sample size in 1401 which is 684 populations (while the number of populations in 2010 is 693). Figure S2: Temporal trends of the global mean and variance of tree growth rates. Calculated using the same data and detrending method as in Figure 1, but with a different moving window length of 3 years (a, b) and 10 years (c, d). Figure S3: Schematic plot showing the averaging process of tree‐level exponents from Taylor's power law models (see Methods for details). Figure S4: Schematic plot showing the analysis to investigate the spatial and temporal regulators of the mean–variance relationship in tree growth rates (see Methods for details). Figure S5: Temporal trends of the mean (a) and variance (b) of tree growth rates in different climate zones. Coloured solid lines are localised smooth regression lines. The thick blue lines denote averages among individual climate zones. Shaded area represents ±1.96 standard error interval. The vertical dashed blue lines denote year 1925 and 1975 for tree growth mean and variance, respectively. Figure S6: Frequency distributions of the p‐value (a) and R 2 (b) for the tree‐level Taylor's power law model. The red line in (b) denotes the median value. Figure S7: Temporal trends of the global mean and variance of tree growth rates. Calculated from tree‐ring series detrended by a 300‐year spline function (see Methods S1). The vertical dashed blue lines denote year 1925 and 1975 for mean and variance, respectively. Shaded area represents ±1.96 stand [file ELE-29-0-s001.docx]

**SUPPLEMENTARY INFORMATION**

**Methods S1: Testing for potential artifacts/biases in tree-ring analysis**

In our analysis we observed significant increases in the mean and variance of tree growth rates over the latest century of the past six hundred years (Fig. 1). To ensure that these results are real, not caused by the artifacts related to common biases arising from different detrending methods, big tree selection bias, slow-grower survivorship bias, changing sample size bias, end bias, age bias, climate sensitivity bias, and presence of multiple series (samples) per tree. In the following sections we analyze these potential sources of biases to test the robustness of our analyses reported in the main text.

1. **Different detrending/standardizing methods**

We tested four major detrending (standardizing) methods that remove intrinsic tree growth trend but retain climate signals in the tree-ring series. They include, as detailed below, negative exponential function (which is used in the main text), spline function detrending, regional curve detrending, and the constant basal area increment detrending. The fourth method, though not often used in dendrochronological analysis, is based on the assumption of constant basal area increment (Biondi & Qeadan 2008), which is supported by some empirical data (Silva et al. 2016; Rupšys 2019).

1. Negative exponential curve: This method fits a negative exponential function (which is the theoretical shape of tree radial growth trend in an unchanging environment) to raw ring-width series and is used to estimate the expected growth rates. This method is especially useful when the climate-driven long-term growth trend is of interest, as it is a conservative detrending method that retains most low-frequency climate signals from raw tree-ring series (Graumlich 1991; Helama et al. 2004; Gedalof & Berg 2010). See Fig. 1.
2. Spline detrending: This method involves fitting a spline function to the raw ring-width series and using it to standardize raw measurements. It has the strength in removing long-term growth signals caused by competition and other non-climatic forces (Cook & Peters 1997). A 32-year spline is most commonly used in previous studies (Helama et al*.* 2004; Babst et al*.* 2019). However, 32-year spline is very flexible and could remove most long-term growth variations driven by exogenous forcing. As climate is typically defined over a period of 30 years, the 32-year spline would inevitably remove the potential long-term tree growth trend related to global warming that we aim to study (Helama et al*.* 2004; Gedalof & Berg 2010). To tackle this problem, here we determined the spline function suitable for our study from the following procedure. We first used and compared six spline curves with wavelengths=32, 100, 200, 300, 400 and 500 years to ‘detrend’ global temperature series (as we did to tree-ring series). We adopted the smallest wavelength that preserved the global temperature trend, which turned out to be 300 years. It means any spline that is more flexible than 300 years (i.e., smaller than 300) would likely remove global warming signal recorded in tree-ring series. We thus used this 300-year spline to test if the global warming signal remained after the tree-ring series were detrended by the spline method (Fig. S7). Besides, we also tested the two-third spline detrending as it is another common detrending method. It applies a spline function with a flexibility (wavelength) that is two-third of the series length. As our tree-ring series had an average length of 150 years, the 2/3 spline on average has a flexibility of around 100 years (Fig. S8).
3. Regional curve standardization: This method estimates an “expected growth rate” for each ring age by averaging all measured ring widths at that age (Helama et al*.* 2004). The expected growth rates are then used to standardize the ring-width series. It requires a large number of tree-ring series covering the full age spectrum in order to obtain a reliable relationship between growth rate and age (Melvin & Briffa 2014). This requirement however is not met by most data in the ITRDB. Besides, this method is also subject to the influence of big tree selection bias and slow-grower survivorship bias (Bowman et al*.* 2013), which we will discuss later. Nonetheless, for the sake of the comparison of detrending methods, we still implemented this regional curve detrending using the *rcs* function in the *dplR* package. See Fig. S9.
4. Constant basal area increment detrending: This method assumes that the annual increment of tree’s basal area is constant. Therefore, the annual ring width could be predicted using a power-function curve. The constant basal area increment detrending involves estimating the expected growth rates by fitting the following model to the raw ring-width series: $w_{t}=\sqrt{\frac{c}{\pi}}\times\left( \sqrt{t}-\sqrt{t-1} \right)$, where *w_t_* is the ring width at age *t*, and *c* is the constant basal area increment. See Fig. S10

The results presented in Fig. S7-S10 consistently show accelerating increase in tree growth variance over the past hundred years, particularly after the 1970’s, regardless of the detrending methods. *R*^2^ values between the variance of the negative exponential method (shown in the main text Fig. 1) and other three methods over the entire study period (1401-2010) vary from 0.54 to 0.87. For the long-term upward trend in mean growth rate, the correlations between the different detrending methods and the negative exponential function are also highly significant (*R*^2^ varying from 0.59 to 0.88) (Fig. S7-S10). It is worth noting that the mean growth rate calculated by 2/3 spline method showed little upward growth trend since the 1900s (Fig. S8). This is anticipated as the two-third spline on average has a flexibility of around 100 years, which should remove most of the long-term growth trend retained by other detrending methods as explained before, regardless of the causes.

As an additional verification to ensure that the observed increasing trends in the mean and variance of tree growth rates with global warming are not caused by detrending bias, we modeled the relationship between raw tree-ring width and temperature using a linear mixed-effects model, controlling for age by including tree age in the random term (random term also includes species and climate zone). The model used individual-level tree growth data and site-level temperature anomalies. The results showed that the effect of temperature was positive and highly significant (*p* < 0.001) for both mean and variance (Table S1), supporting the main findings in Fig. 1. However, because tree age cannot be accurately determined for the ITRDB dataset (we can only estimate tree age by counting the rings, which involve much uncertainty as earliest rings are often missing in tree-ring samples), we did not use this approach in the main analysis and instead relied on the modified negative exponential detrending approach.

1. **Big tree selection bias and slow-grower survivorship bias**

Big tree selection bias and slow-grower survivorship bias are two most common biases in tree-ring analyses. They both lead to an upward growth trend over time. Big tree selection bias occurs when tree-ring sampling only targets trees with stem diameter larger than a certain threshold (>30 cm, for example), which is a common practice in tree-ring study. In such a case, the data set would be increasingly represented by fast-growing trees in recent years, as fast-growing trees are more likely to grow large to be included in the sample (Bowman et al*.* 2013). This would result in a false upward growth trend in the average ring-width chronology. In a similar way, slow-grower survivorship bias could also occur. In tree-ring data set, trees with rings of earlier years are older. In other words, trees germinated earlier need to live older to be sampled. In general, slow-growing trees often live longer than the fast-growing trees (Bowman et al*.* 2013; Brienen et al. 2020). Therefore, measurements for earlier years are more likely to come from slow-growing trees, resulting in an apparent upward growth trend in the average ring-width over time (Bowman et al*.* 2013). However, it has been recognized that these two biases do not affect average growth trend calculated from individually-detrended tree-ring series, because the difference in mean growth rates among fast and slow-growing trees has been eliminated in the standardization process (Bowman et al*.* 2013; Duchesne et al*.* 2019). This conclusion should also apply to this study because our tree-ring series were individually detrended.

1. **Changing sample size bias**

This bias could be a real concern as the sample size of our study constantly changed over the study period, especially after the 1990s and before the 1600’s, when it is much reduced (Fig. S1). This means that if we compare the global average tree growth patterns (either mean or variance) among different time periods, we are actually comparing the values calculated from different subsets of trees of the whole data set. Therefore, an observed tendency in the global average could be confounded by the change in different subsets of trees, possibly biasing the real tree growth patterns. To address this issue, we recalculated global tree growth patterns using the 23723 ring-width series that completely covered 1801-2000 (the period selected as a balance between the temporal coverage and sample size of the qualified series). This data allowed us to test if significant increases in both mean and variance can still be observed. The result shows that, after the sample size being controlled, the increasing trends in both mean and variance remain pronounced (Fig. S11) and are similar to those shown in Fig. 1 (with *R*^2^>0.93), thus ruling out the possible bias of changing sample size.

1. **End bias**

As shown in Fig. 1, we found a steep increase in global variance of tree growth rates in the past century, particularly after the 1970s. However, this period of time is also a period when most tree-ring series ended. Studies have shown that tree growth variation could increase before the death of a tree (Cailleret et al*.* 2019). This means that for tree-ring series taken from trees approaching death, the growth variation could increase at the end of the series. Moreover, as trees grow older, their ring widths tend to become smaller, which could lead to more fluctuating measurements and a lack of fit in detrending process (Cook & Peters*.* 1997). The former directly increases growth variance, and the latter could lead to unrealistically high (or low) RWI values, subsequently affect growth variance. To test if these factors might cause the observed surge in tree growth variance, we first removed trees with any RWI value >10 (which is suspiciously high) in any year before averaging them into the population-level and subsequently global values. This step excluded ~0.2% trees. The results show that the overall shape and magnitude of the post-1970s variance increase remained almost identical to those reported in the main text (a 40% vs. 41% increase). This suggests that the high RWI values (possibly caused by end bias) were unlikely the main driver of the reported variance surge.

In addition, we further recalculated the global average 5-year variance using all the tree-ring series (*n*=44331) that ended by 1970. The idea is that if the end bias were the cause for the increasing variance observed in Fig. 1, we would also expect a similar spurt in variance in the decades before 1970 using this new data set. However, this is not the case as shown in Fig. S12. When tree-ring series before 1970 were used, there was a mild and gradual increase in growth variance (~13% increase in 1941-1970 compared to in 1401-1940), but its amplitude and abruptness were much smaller than those shown in Fig. 1b. This result suggests there may be a moderate end bias but the sudden increase in post-1970s variance observed in our main result (Fig. 1b) was unlikely to be primarily driven by it.

Here we also tested if end bias may contribute to the upward trend in mean growth rate starting in the early 1900’s as shown in Fig. 1a. A potential cause of this end bias could be competition by which trees growing in a closed-canopy environment experience a “suppression-release” growth when forest gaps are formed (Cho & Boerner 1995). In this case, the negative exponential detrending may underestimate the “expected growth rate” for rings formed after growth is released, leading to an increasingly overestimated RWI towards the end of the series. Although this unlikely occurred in our data as we observed that the increase of global mean RWI actually slowed down after the mid-20^th^ century (Fig. 1), we still tested it using the following method. To do that, we recalculated the average growth trend but restricted the analysis to the 30413 series that ended by 1900. If there was an increasing growth signal caused by growth release as described above, we would expect a similar growth increase near 1900. But as shown in Fig. S12, this was not observed. Instead, the mean growth rate had a *R*^2^ of 0.84 with that in Fig. 1 for the same period, showing the robustness of our detrending method against this potential bias.

1. **Age bias**

Tree growth variance could also increase as trees grow older due to aging process. To ensure that the sudden increase in growth variance after the 1970’s shown in Fig. 1 was not caused by the aging of trees, we repeated the reconstruction of the global history of tree growth variance by controlling tree age. This was done by calculating the global tree growth variance using the data from trees within a narrow age range. For example, when calculating the global variance for time window 1971-1975 and for age group 1-20 years, we only used the trees at 1-20 ring age in 1975. We did this for a total of 10 age groups: 1-20, 21-40, … up to 181-200 years old. Age was determined by counting the number of rings presented in the series (Gunnarson et al*.* 2011; Brienen et al*.* 2020). This analysis ensures that any observed trend in the growth variance was not caused by the factor of aging, as the average tree age has been fixed over time. The result of this analysis is presented in Fig. S13 which shows that after tree age being controlled, trees of different age groups had a very similar temporal increase in growth variance as that in Fig. 1 (*R*^2^ varies from 0.39 to 0.97, with mean *R*^2^=0.66), ruling out the age bias.

1. **Climate sensitivity bias**

The ITRDB was originally created to archive tree-ring data for the purpose of climate reconstructions, or dendroclimatological study. Because of that, the indiscriminate use of the data to studies other than dendroclimatology, such as modeling tree growth rates and variance, could potentially be subject to the so-called “climate sensitivity bias” (Babst et al. 2019). This is because in dendroclimatological study target trees are selected to maximize the time span and climate signals embedded in the ring-width series, making them less representative of younger trees and trees growing in less-restricted environment. This could cause overestimation of climate sensitivity and growth variations under climate change in general. To test this bias, several ecology-oriented studies have compared the ITRDB data with regional forest-census data that are considered more representative of the whole tree population, e.g., the Canadian National Forest Inventory (NFI) data (Babst et al. 2019) and the American Forest Inventory and Analysis (FIA) data (Klesse et al. 2018). These tests indicate that the climate sensitivity bias, even exists, does not change the general growth patterns and their relationships with climate (Klesse et al. 2018; Babst et al*.* 2019). These findings are commonly cited to justify the use of the ITRDB data (Zuidema et al. 2022). However, using regional forest-census data to ‘benchmark’ the ITRDB dataset may still not be sufficient for making inferences on the global scale. In this study, we went one step further to verify the potential climate sensitivity bias. We noticed that, in the ITRDB, some of the datasets were compiled from studies that were not intended for dendroclimatological study but for other more general dendrochronological research. We therefore classified the source studies into two categories: dendroclimatology-oriented studies and other studies. The first category includes studies using tree-ring as a proxy to reconstruct past climates, which is often considered subject to the climate sensitivity bias. The second category includes studies using tree-ring to investigate broader topics like tree ecology and carbon dynamics, which emphasize data representativity in sampling and are considered less subject to the climate sensitivity bias. In total, we identified 109 dendroclimatological studies and 100 non-dendroclimatological studies, accounting for 952 and 718 population-level datasets, respectively (Table S2: Grouped_Studies.csv). The median tree age in year 1960 (the year with the highest sample depth) was 175 years for the dendroclimatology group, and 77 for the non-dendroclimatology group, which had a similar age difference as in Klesse et al. (2018) who used FIA dataset as a non-dendroclimatology benchmark. This age difference also confirms the general belief that data from dendroclimatology studies tend to be over-represented by older trees. We recalculated the mean tree growth rate and variance for each of the two groups. As presented in Fig. S14, the historical trajectories of both quantities show no qualitative difference between the two groups and closely match those using the whole ITRDB dataset in Fig. 1, with *R*^2^≥0.69 for the non-dendroclimatological group to ≥0.83 for the dendroclimatological group over the entire study period of 1401-2010. If only the period of the recent 200 years (1801-2010) in which the sample size was much increased compared to earlier years, the *R*^2^ was larger than 0.87 for both groups. This confirms that the rapid increase in tree growth variation after the 1970s was not caused by the over-representation of dendroclimatological study bias. The ITRDB dataset is suitable for our study.

1. **Presence of multiple series (samples) per tree**

In tree-ring sampling, multiple cores per tree are sometimes collected for reasons like capturing uneven tree growth in different directions or using as backup samples. They are sometimes all presented in the final dataset, leading to the presence of multiple tree-ring series for the same tree. In such cases, the last character of the series ID is commonly used to identify cores, e.g., xxxxx1, xxxxx2, or xxxxxA, xxxxxB. But this is not standardized and different researchers may label ring-width series differently. For the ITRDB, it does not provide such information to identify multiple cores. The IDs of individual series in the ITRDB are referred to as “TreeID”. Therefore, technically we are supposed to treat them as individual trees. That may be the reason why most studies do not distinguish between series and trees. However, we could still use our best knowledge to test whether this issue has potentially biased the growth patterns. To do so, we selected tree-ring series with “TreeID” that was unique even after the last character was removed. For this group of data, we were confident that the selected series should each represent one unique tree (representing 25% series and 74% populations of the whole data set). We then grouped the rest of the data (i.e., trees that might have multiple series, accounting for 75% series and 89% populations) to calculate the mean growth rates and variance. As shown in Fig. S15, the growth trends of the two groups are almost identical to Fig. 1 (*R*^2^>0.96). This suggests that multiple cores, if even existing, do not cause systematic bias in the observed growth patterns. In addition, we would like to point out that, the presence of multiple cores per tree did not inflate our sample size or bias the estimation of confidence intervals or *p­*-values, because these statistics were estimated from population-level values rather than on the individual level (i.e., the sample size was the number of populations, not individual series, see Methods).

1. Cho, D. S., & Boerner, R. E. J. (1995). Dendrochronological analysis of the canopy history of two Ohio old-growth forests. *Vegetatio*, 120, 173-183.
2. Cook, E. R., & Peters, K. (1997). Calculating unbiased tree-ring indices for the study of climatic and environmental change. *The Holocene*, 7(3), 361-370.
3. Graumlich, L.J. (1991) Subalpine tree growth, climate, and increasing CO_2_: an assessment of recent growth trends. *Ecology*, 72, 1-11.
4. Gunnarson, B. E., Linderholm, H. W., & Moberg, A. (2011). Improving a tree-ring reconstruction from west-central Scandinavia: 900 years of warm-season temperatures. *Climate Dynamics*, 36, 97-108.
5. Melvin, T. M., & Briffa, K. R. (2014). CRUST: software for the implementation of regional chronology standardisation: part 1. Signal-free RCS. *Dendrochronologia*, 32(1), 7-20.
6. Rupšys, P. (2019) Modeling dynamics of structural components of forest stands based on trivariate stochastic differential equation. *Forests*, 10, 506.
7. Zuidema, P. A., Babst, F., Groenendijk, P., Trouet, V., Abiyu, A., Acuña-Soto, R., ... & Zhou, Z. K. (2022). Tropical tree growth driven by dry-season climate variability. *Nature* *Geoscience*, 15(4), 269-276.

**TABLE S1.** Linear mixed-effects model for the responses of temporal mean and variance of tree growth rate (in mm) to temperature. Coefficient (se) indicates the effect (slope) of a predictor and its related standard error.

| **Response variable** | **Predictor** | **Coefficient (se)** | ***p-*value** |
| --- | --- | --- | --- |
| Mean | MAT | 0.23 (7.2E-03) | 2.2E-32 |
| Variance |  | 0.11 (3.8E-04) | 1.3E-56 |

**FIGURE S1**


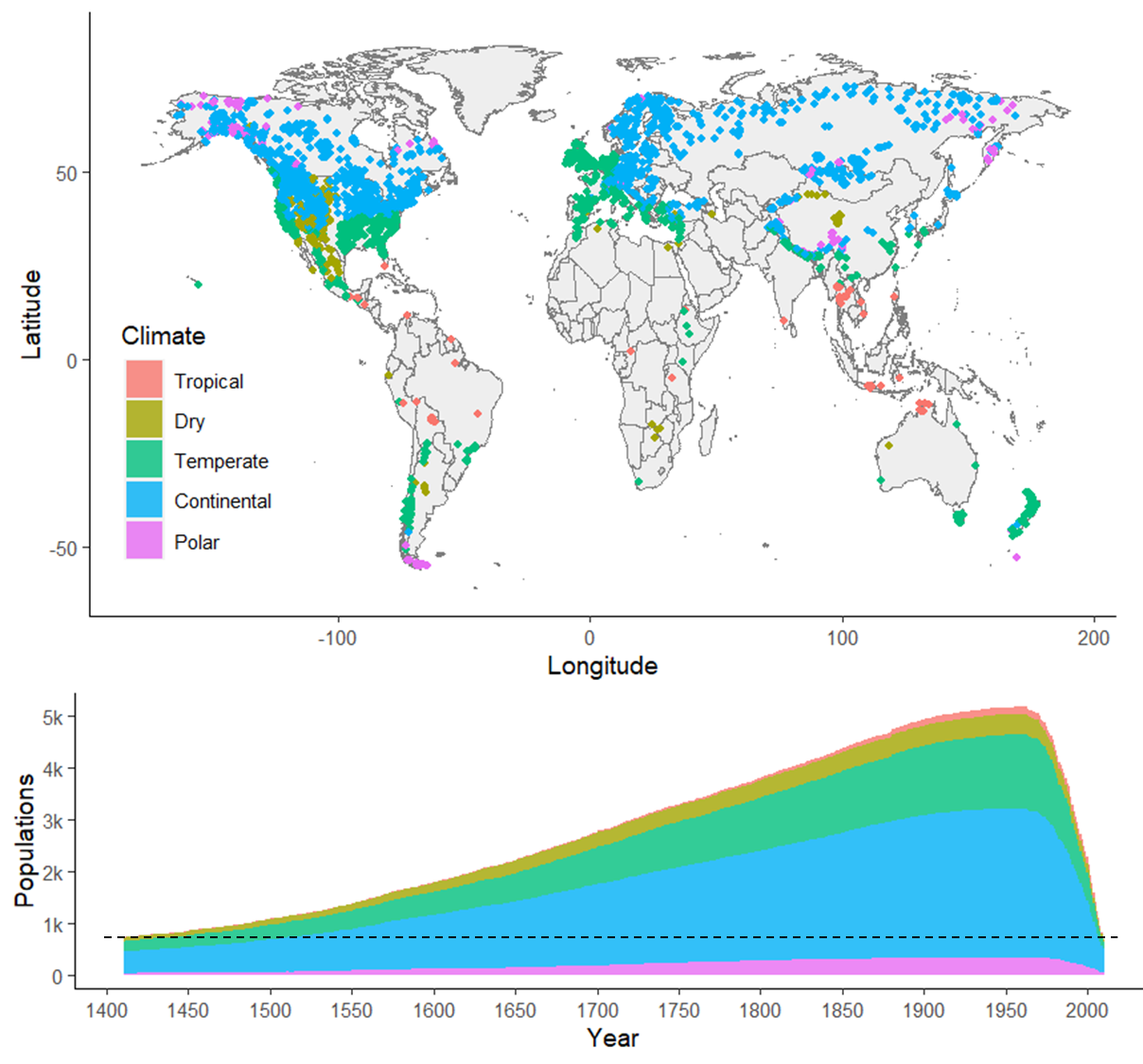


**FIGURE S1**. Geographical distribution (upper panel) and sample size (lower panel) of the tree-ring data set. In the upper panel, colors denote different Köppen climate zones. In the lower panel the *y* axis denotes number of populations. The black horizontal line denotes the sample size in 1401 which is 684 populations (while the number of populations in 2010 is 693).

**FIGURE S2**


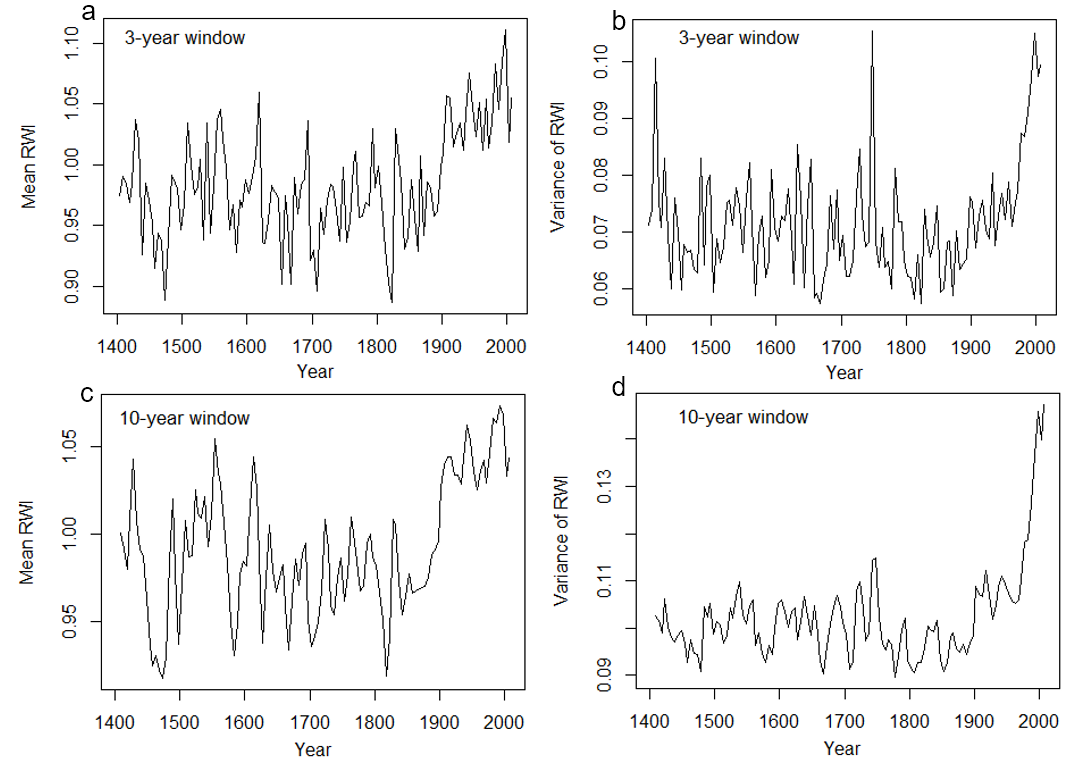


**FIGURE S2.** Temporal trends of the global mean and variance in tree growth rates. Calculated using the same data and detrending method as in Fig. 1, but with a different moving window length of 3 years (a, b) and 10 years (c, d).

**FIGURE S3**


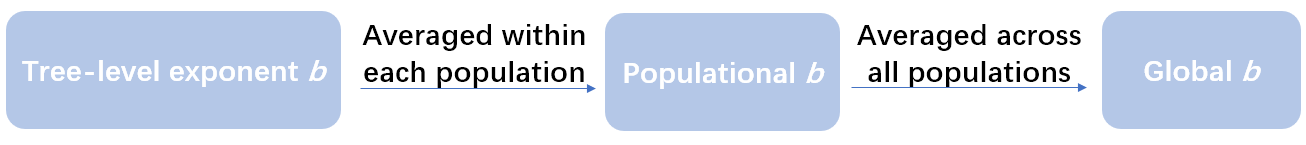


**FIGURE S3.** Schematic plot showing the averaging process of tree-level exponents from the Taylor’s power law models (see Methods for details).

**FIGURE S4**


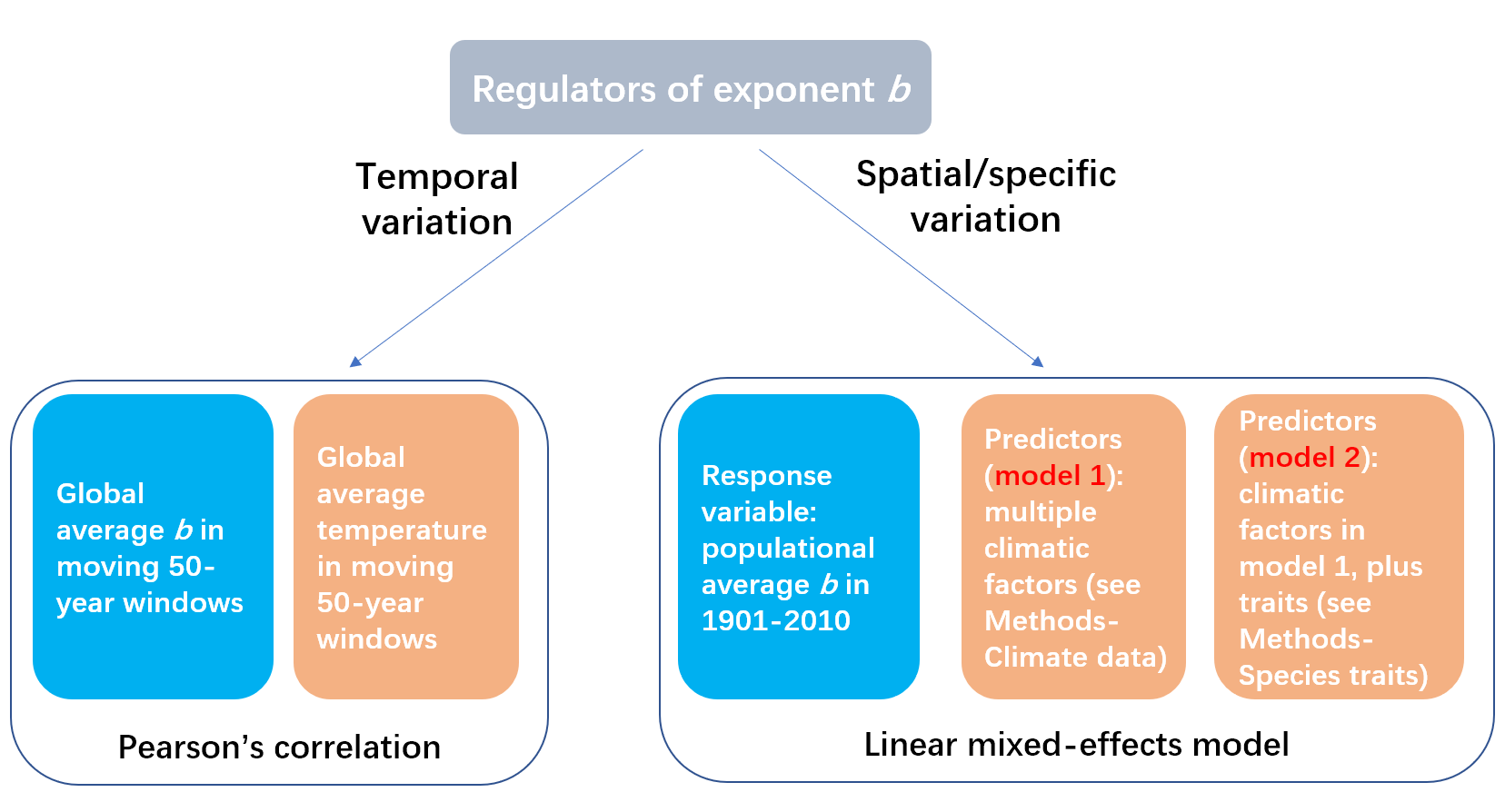


**FIGURE S4.** Schematic plot showing the analysis to investigate the spatial and temporal regulators of the mean-variance relationship in tree growth rates (see Methods for details).

**FIGURE S5**


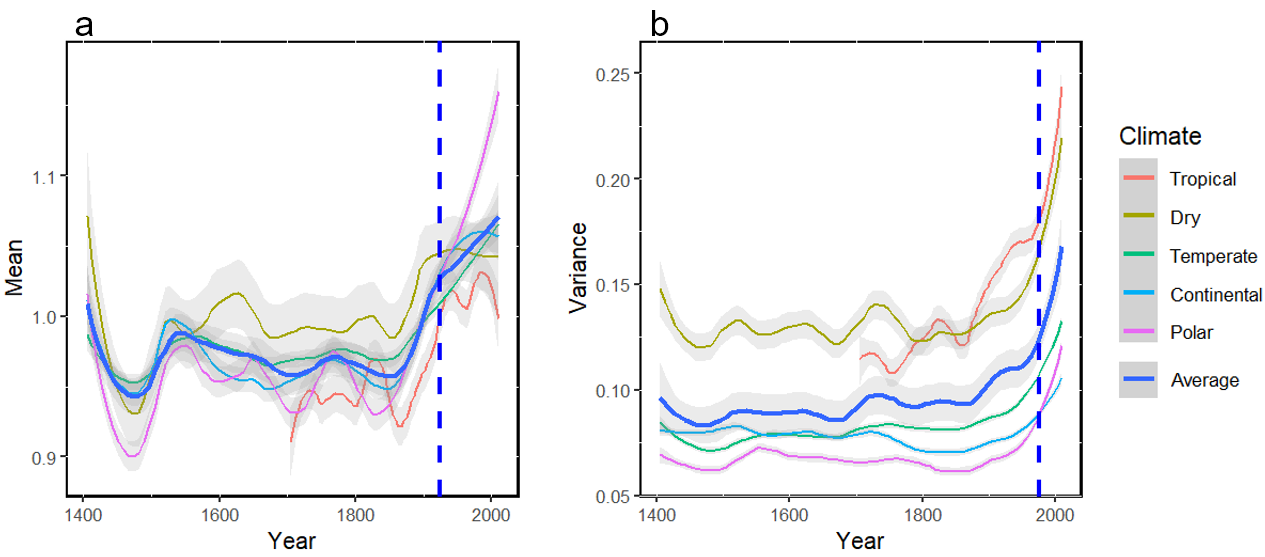


**FIGURE S5.** Temporal trends of the mean (a) and variance (b) of tree growth rates in different climate zones. Colored solid lines are localized smooth regression lines. The thick blue lines denote averages among individual climate zones. Shaded area represents ±1.96 standard error interval. The vertical dashed blue lines denote year 1925 and 1975 for tree growth mean and variance, respectively.

**FIGURE S6**


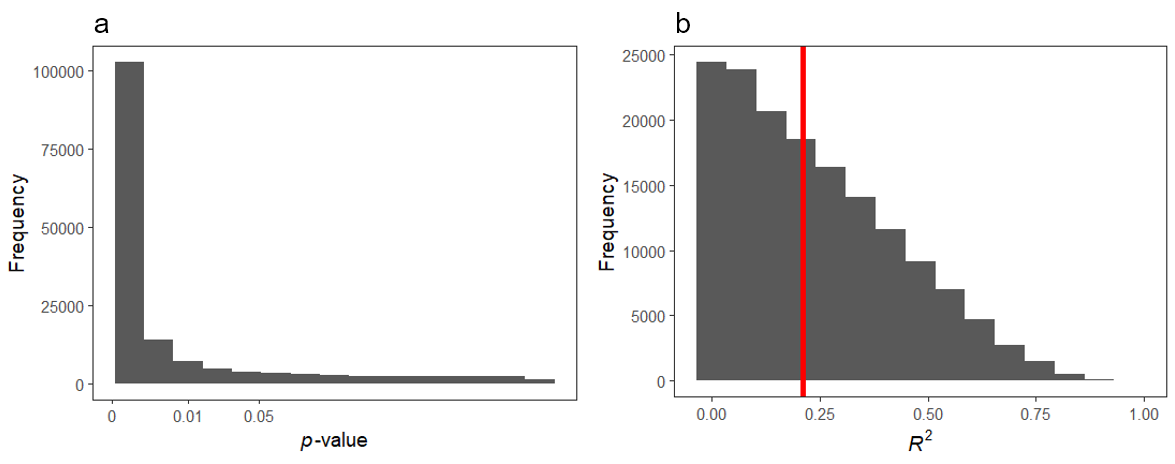


**FIGURE S6.** Frequency distributions of the *p*-value (a) and *R*_­_^2^ (b) for the tree-level Taylor’s power law model. The red line in (b) denotes the median value.

**FIGURE S7**


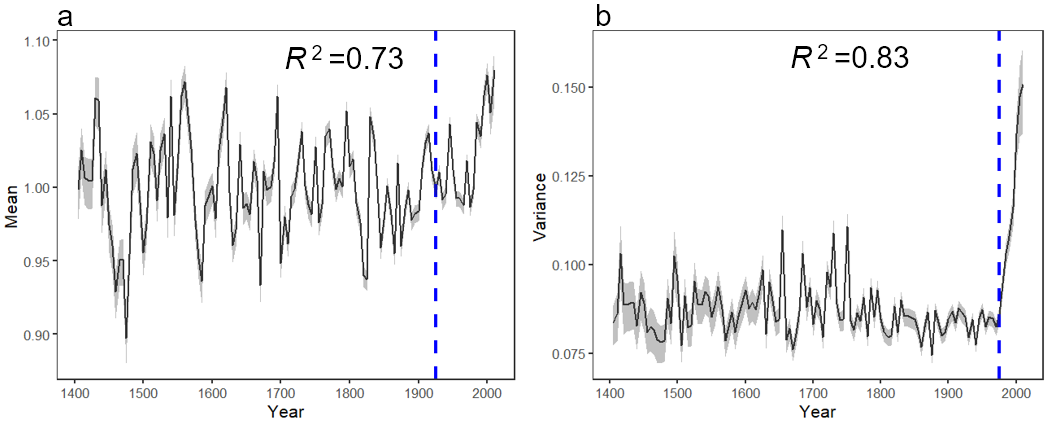


**FIGURE S7**. Temporal trends of the global mean and variance in tree growth rates. Calculated from tree-ring series detrended by a 300-year spline function (see Methods S1). The vertical dashed blue lines denote year 1925 and 1975 for mean and variance, respectively. Shaded area represents ± 1.96 standard error interval. The *R*^2^ values show the correlation between the growth mean or variance and that in Fig. 1.

**FIGURE S8**


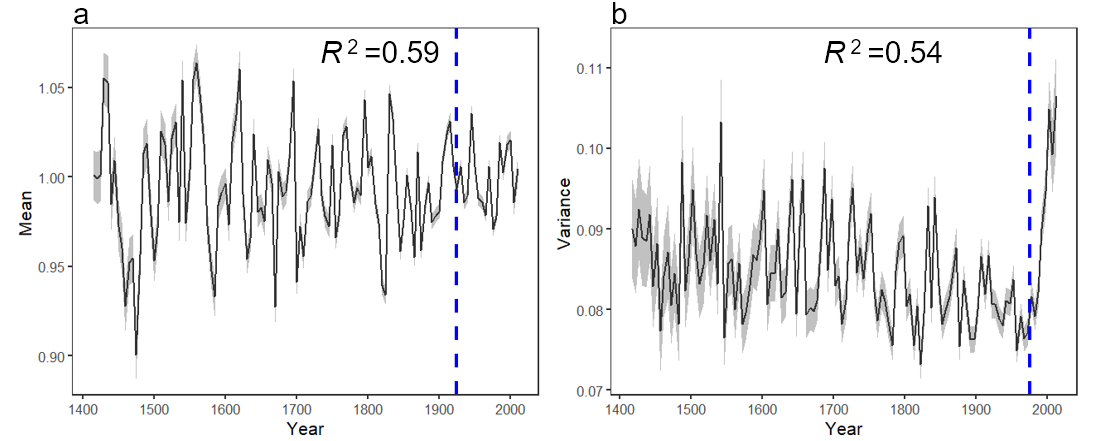


**FIGURE S8**. Temporal trends of the global mean and variance in tree growth rates. Calculated from tree-ring series detrended by the 2/3 spline function. The vertical dashed blue lines denote year 1925 and 1975 for mean and variance, respectively. Shaded area represents ± 1.96 standard error interval. The *R*^2^ values show the correlation between the growth mean or variance and that in Fig. 1.

**FIGURE S9**


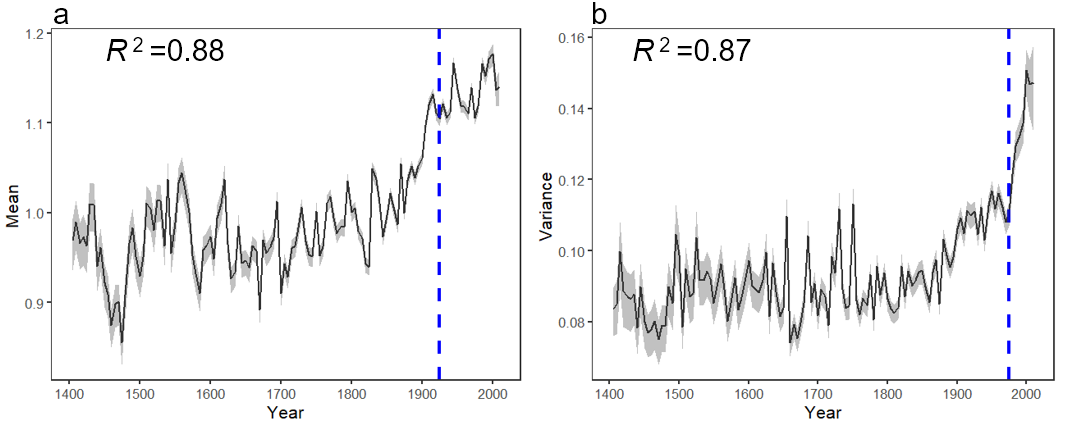


**FIGURE S9**. Temporal trends of the global mean and variance in tree growth rates. Calculated from tree-ring series detrended by the regional curve standardization. The vertical dashed blue lines denote year 1925 and 1975 for mean and variance, respectively. Shaded area represents ± 1.96 standard error interval. The *R*^2^ values show the correlation between the growth mean or variance and that in Fig. 1.

**FIGURE S10**


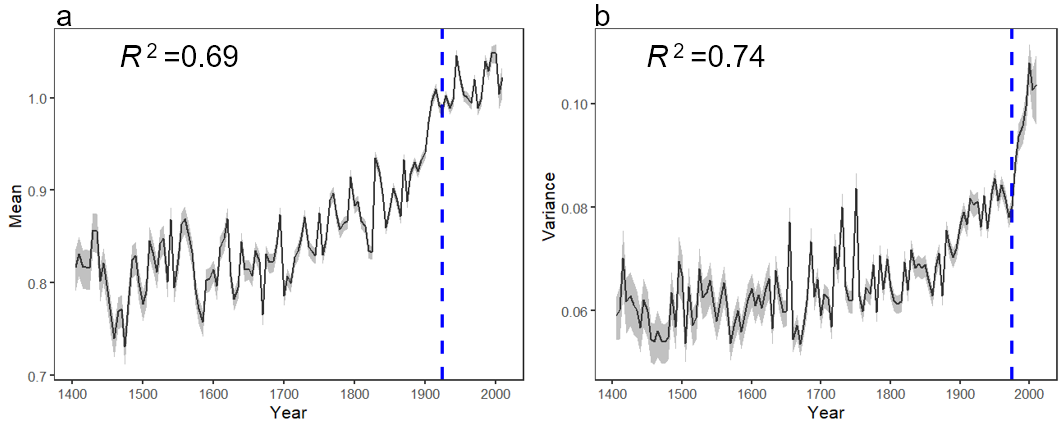


**FIGURE S10**. Temporal trends of the global mean and variance in tree growth rates. Calculated from tree-ring series detrended by constant basal area increment detrending. The vertical dashed blue lines denote year 1925 and 1975 for mean and variance, respectively. Shaded area represents ± 1.96 standard error interval. The *R*^2^ value shows the correlation between the growth mean or variance and that in Fig. 1.

**FIGURE S11**


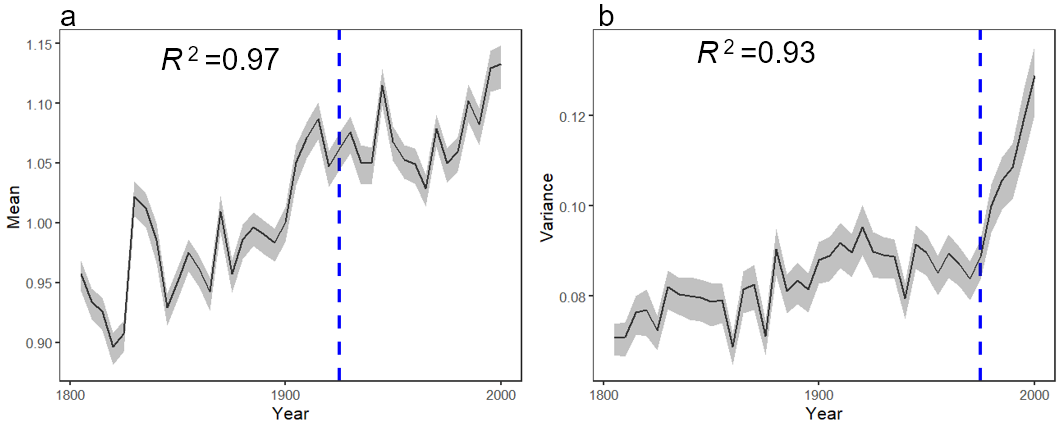


**FIGURE S11**. Temporal trends of the global mean and variance in tree growth rates. Calculated from tree-ring series fully covering 1801-2000 (no change in data coverage and sample size over this 200-year period). The vertical dashed blue lines denote year 1925 and 1975 for mean and variance, respectively. Shaded area represents ± 1.96 standard error interval. The *R*^2^ values indicate the correlation between the growth mean or variance and that in Fig. 1 in the same period of coverage time (1801-2000).

**FIGURE S12**


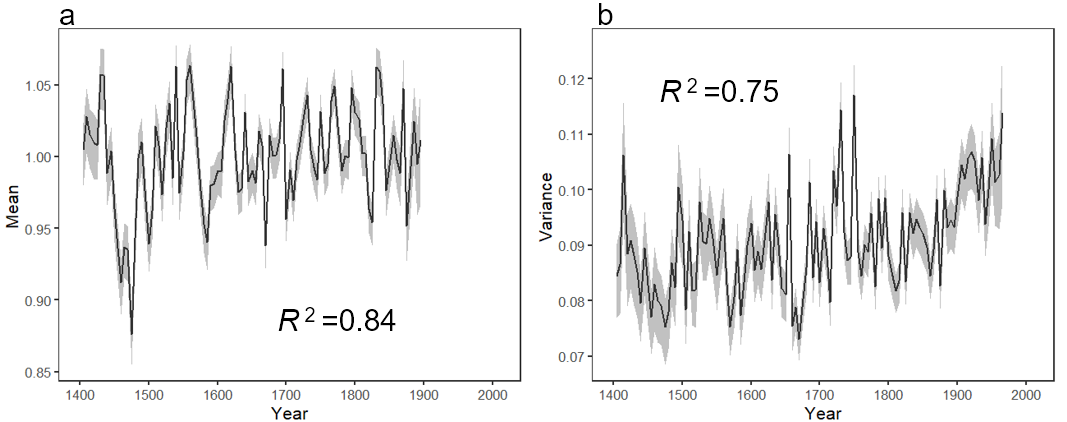


**FIGURE S12**. Temporal trends of the global mean and variance in tree growth rates. Calculated from tree-ring series end before 1900 (for mean) and 1970 (for variance) (see Methods S1). Shaded area represents ± 1.96 standard error interval.

**FIGURE S13**


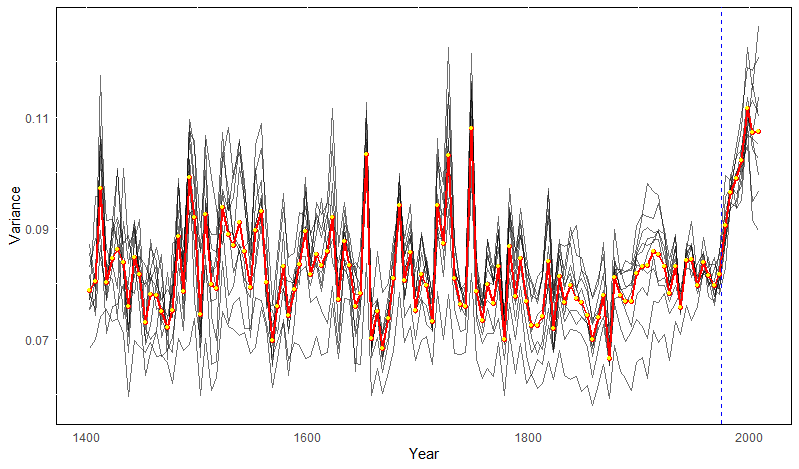


**FIGURE S13**. Temporal trends of the global variance in tree growth rates. The variance was calculated for different age groups of trees separately. Each curve represents one age group. There are 10 groups in total. The red line denotes average values across all groups and each yellow point represents one 5-year window over which growth variance was calculated. The vertical dashed blue line denotes year 1975.

**FIGURE S14**


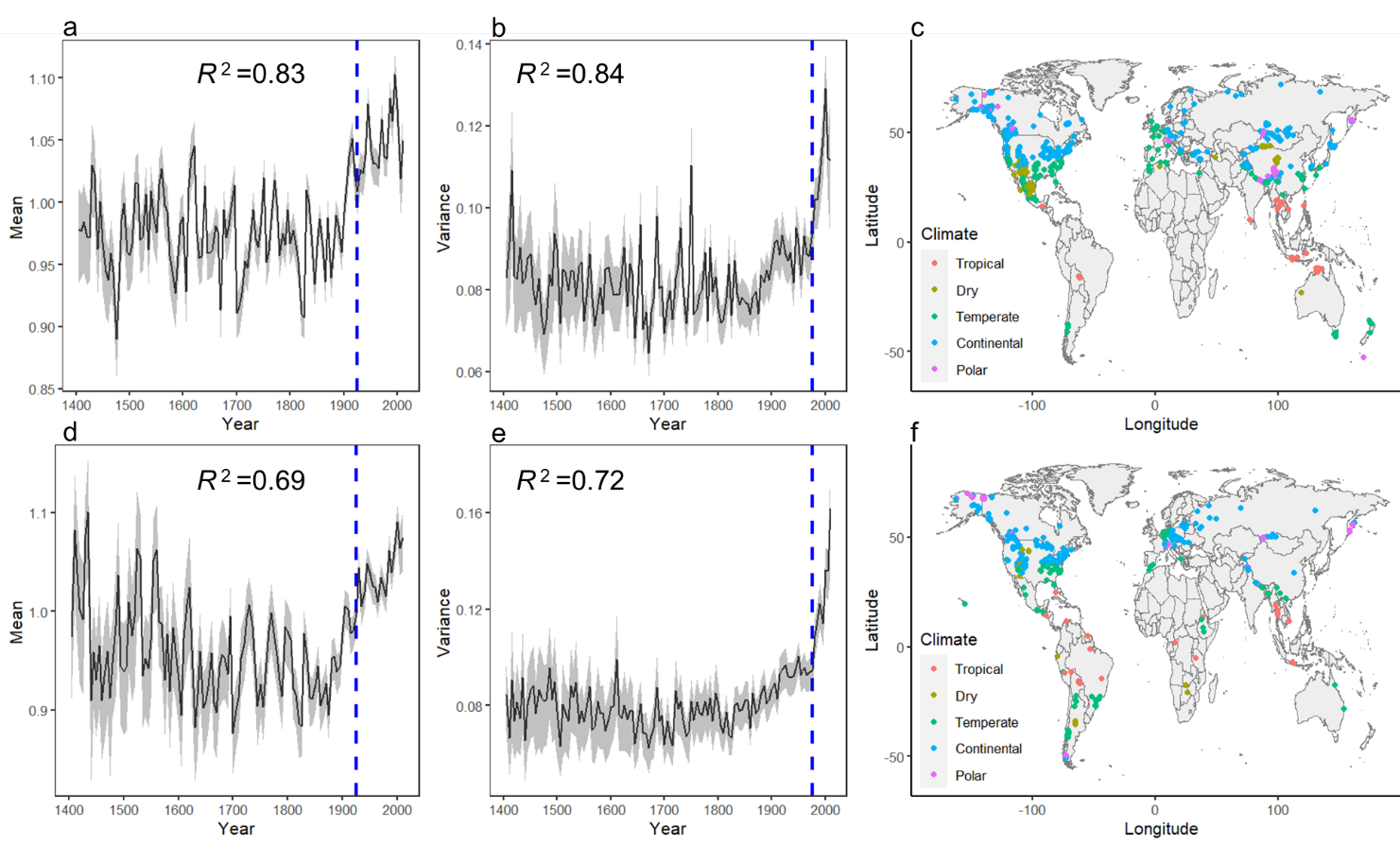


**FIGURE S14**. Temporal trends of the global mean and variance in tree growth rates. Calculated from the ITRDB tree-ring data that were originally collected for climate-reconstruction studies (*upper panels*) and for other more general dendrochronological studies *(lower panels*). The global distributions of the two groups are displayed on the maps on the right, showing a fair global coverage. The vertical dashed blue lines denote year 1925 and 1975 for mean and variance, respectively. Shaded area represents ± 1.96 standard error interval. The *R*^2^ values show the correlation of the mean growth rate and variance with those in Fig. 1.

**FIGURE S15**


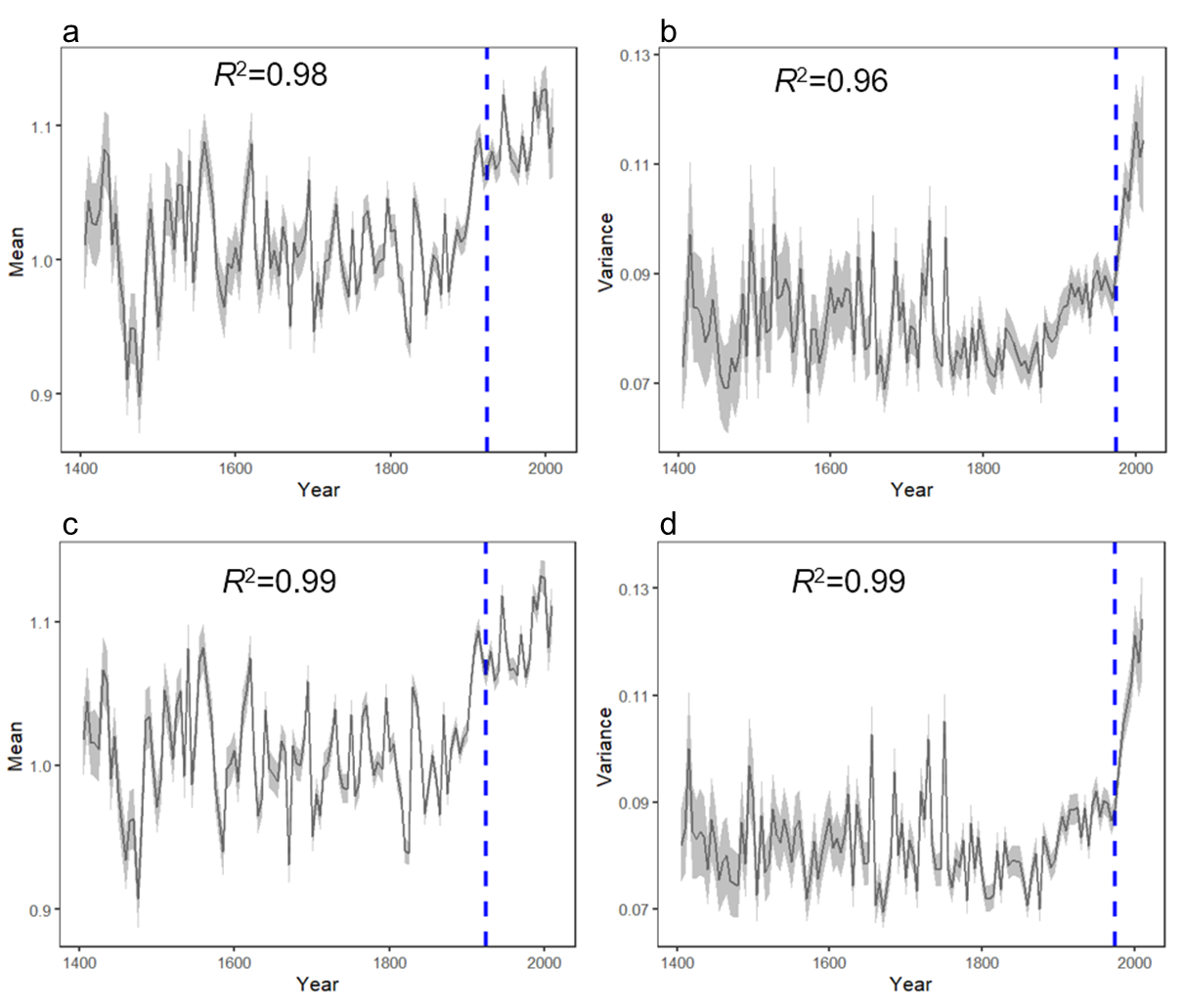


**FIGURE S15**. Temporal trends of the global mean and variance of tree growth rates. Calculated from the group of trees represented by single series (*upper panels*) and the group of trees that may be represented by multiple series (*lower panels*). The vertical dashed blue lines denote year 1925 and 1975 for mean and variance, respectively. Shaded area represents ± 1.96 standard error interval. The *R*^2^ values show the correlation of the mean growth rate and variance with those in Fig. 1.
